# HSP90-incorporating chaperome networks as biosensor for disease-related pathways in patient-specific midbrain dopamine neurons

**DOI:** 10.1038/s41467-018-06486-6

**Published:** 2018-10-19

**Authors:** Sarah Kishinevsky, Tai Wang, Anna Rodina, Sun Young Chung, Chao Xu, John Philip, Tony Taldone, Suhasini Joshi, Mary L. Alpaugh, Alexander Bolaender, Simon Gutbier, Davinder Sandhu, Faranak Fattahi, Bastian Zimmer, Smit K. Shah, Elizabeth Chang, Carmen Inda, John Koren, Nathalie G. Saurat, Marcel Leist, Steven S. Gross, Venkatraman E. Seshan, Christine Klein, Mark J. Tomishima, Hediye Erdjument-Bromage, Thomas A. Neubert, Ronald C. Henrickson, Gabriela Chiosis, Lorenz Studer

**Affiliations:** 1 The Center for Stem Cell Biology, Memorial Sloan Kettering Cancer Center, 1275 York Avenue, Box 256, New York, NY 10065 USA; 2 Developmental Biology Program, Memorial Sloan Kettering Cancer Center, 1275 York Avenue, Box 256, New York, NY 10065 USA; 3 Program in Chemical Biology, Memorial Sloan Kettering Cancer Center, 1275 York Avenue, New York, NY 10065 USA; 4 Neuroscience Graduate Program of Weill Cornell Graduate School of Biomedical Sciences, Weill Cornell Medical College, 1300 York Avenue, Box 65, New York, NY 10065 USA; 5 Proteomics Core Facility, Memorial Sloan Kettering Cancer Center, 1275 York Avenue, New York, NY 10065 USA; 6 Doerenkamp-Zbinden Chair for In Vitro Toxicology and Biomedicine, University of Konstanz, Konstanz, 78464 Germany; 7 Department of Pharmacology, Weill Cornell College of Medicine, 1300 York Avenue, New York, NY 10065 USA; 8 Department of Epidemiology and Biostatistics, Memorial Sloan Kettering Cancer Center, New York, NY 10017 USA; 9 Institute of Neurogenetics, University of Lübeck, Lübeck, 23538 Germany; 10 SKI Stem Cell Research Facility, 1275 York Avenue, Sloan Kettering Institute, New York, NY 10065 USA; 11 Department of Cell Biology, NYU School of Medicine, New York, NY 10016 USA; 12 Kimmel Center for Biology and Medicine at the Skirball Institute, NYU School of Medicine, New York, NY 10016 USA; 13 Department of Medicine, Memorial Hospital, Memorial Sloan Kettering Cancer Center, 1275 York Avenue, New York, NY 10065 USA; 14 Department of Molecular and Cellular Biosciences, Rowan University, 1275 York Avenue, Glassboro, NJ 08028 USA; 15 Hostos Community College, City University of New York, Bronx, NY 10453 USA; 16 Department of Biochemistry, University of Notre Dame, Notre Dame, IN 46556 USA

## Abstract

Environmental and genetic risk factors contribute to Parkinson’s Disease (PD) pathogenesis and the associated midbrain dopamine (mDA) neuron loss. Here, we identify early PD pathogenic events by developing methodology that utilizes recent innovations in human pluripotent stem cells (hPSC) and chemical sensors of HSP90-incorporating chaperome networks. We show that events triggered by PD-related genetic or toxic stimuli alter the neuronal proteome, thereby altering the stress-specific chaperome networks, which produce changes detected by chemical sensors. Through this method we identify STAT3 and NF-κB signaling activation as examples of genetic stress, and phospho-tyrosine hydroxylase (TH) activation as an example of toxic stress-induced pathways in PD neurons. Importantly, pharmacological inhibition of the stress chaperome network reversed abnormal phospho-STAT3 signaling and phospho-TH-related dopamine levels and rescued PD neuron viability. The use of chemical sensors of chaperome networks on hPSC-derived lineages may present a general strategy to identify molecular events associated with neurodegenerative diseases.

## Abstract (web summary)

The early molecular events that ultimately lead to neuronal cell death in pathologies such as Parkinson’s disease are poorly understood. Here the authors use pluripotent stem-cell-derived human midbrain neurons and chemical biology tools to gain molecular level insight into the events induced by toxic and genetic stresses that mimic those occurring during neurodegeneration.

## Introduction

A large percentage of midbrain dopamine (mDA) neurons is permanently lost by the time the clinical diagnosis of Parkinson’s disease (PD) is made^1^. This is a major challenge for the identification of early disease events and an impediment to the development of disease-modifying therapeutic strategies. While a minority of PD cases can be attributed to a defined genetic cause, the majority are thought to be triggered by a combination of genetic and environmental risk factors^2^. Recent advances in generating patient-specific pluripotent stem cells (PSCs) and PSC-derived mDA neurons^3^ make it possible to examine how genetic and environmental stressors induce early PD pathogenic events. For example, we and others have previously shown that *Parkin*- or *PINK1*-mutant PSC-derived mDA neurons display Parkinsonian phenotypes in vitro^4^ and after transplantation in vivo, especially upon induction of age-associated stress^5^. Parkin and PINK1 interact with each other during mitophagy^6,7^, and mutations in either of those two genes are linked to early onset PD^8–14^.

In neurodegenerative disease such as PD, it has been hypothesized that heat shock protein 90 (HSP90) may facilitate pathogenic events by stabilizing disease-related proteins and preventing their degradation^15–20^. Moreover, HSP90 and other chaperones accumulate in Lewy bodies of PD patient brains^21^. HSP90 co-chaperone complexes are critical for folding client proteins under basal housekeeping conditions^22^, and refolding denatured proteins in response to endogenous and exogenous stress pressures^23,24^. Cellular stress, either genetic or environmental, may trigger a shift in the balance of housekeeping HSP90 (H-HSP90) activities towards thermodynamically distinct pools of stress HSP90 (S-HSP90), thereby supporting altered protein networks^24–28^. In cancer cells, we have demonstrated that S-HSP90 is stably engaged with a number of other chaperones, co-chaperones, isomerases, and scaffolding and adaptor proteins^25,27^, players collectively referred to as the chaperome^29^.

Disease states are often associated with changes in chaperomes such as those driven by alterations in the expression level of chaperome members. Landmark studies have demonstrated how changes in chaperome subnetworks are associated with aging and neurodegenerative diseases^29,30^ and may signify a collapse in the chaperome folding machinery, permissive of protein aggregate formation, a hallmark of many neurodegenerative diseases, including PD. Accordingly, depletion of subsets of molecular chaperones during the progression of PD may exacerbate protein toxicity and neurodegeneration^31,32^.

Changes in chaperomes due to alterations in the interaction strength between the participant proteins have also been reported^27,28^. In cancer cells, the chaperome may form complexes of enhanced stability that bring together the chaperome units into the formation of stable chaperome networks better suited to deal with the proteome demand present in the malignant state. These chaperome entities, thermodynamically and functionally distinct from constituent chaperomes, have been termed epichaperomes. In contrast, in healthy cells chaperome units are present in dynamic complexes or in a non-complexed form^27,33^. In this view chaperome function and structural organization, but not necessarily levels, are modulated by cellular stress associated with malignancy, and the goal of such chaperome restructuring may be to increase cellular adaptation by augmenting the fitness or protein networks and pathways^28^. How changes in the interaction strength between chaperome members in PD influence chaperome networks and proteome function remains unknown.

We here query whether the use of patient-specific PSC-derived mDA neurons and PD-related stress conditions in combination with chemical probes of chaperome networks can be used to identify and characterize early PD-related proteome alterations, and to understand whether, and how, such stresses remodel the chaperome networks in PD. Supplementary Fig. 1 outlines our hypothesis and experimental approach. Our method makes use of PU-H71, an HSP90 inhibitor that displays higher affinity for HSP90 when HSP90 is part of the stable chaperome complexes of chaperome networks formed under stress (i.e., epichaperomes)^25,27^. Therefore, PU-H71 and related chemical probes can be used as chemical sensors to recognize the stress-modified HSP90 pool incorporated into chaperome networks, and moreover, capture and identify the disease-related protein networks it regulates (reviewed by refs. ^24^^,34^). We here use these chemical probes to provide proof-of-principle on how stress alters HSP90-associated chaperome networks in PD and to assess the consequences such changes have on the PD proteome.

## Results

### HSP90 complexes in hPSCs and hPSC-derived mDA neurons

We recently demonstrated that production of highly enriched dopamine neurons, of good quality and in a quantity useful for disease investigation, depends on the type of differentiation protocol utilized. Using a floor-plate-based but not a neural-rosette-based directed differentiation strategy, yields midbrain neurons that express key mDA markers including the transcription factors FOXA2 and LMX1A. These neurons efficiently engraft in vivo, and can restore amphetamine-induced rotation behavior and induce improvements in tests of forelimb use and akinesia in multiple PD animal models^3^. The iPSC-derived mDA neurons recapitulate PD phenotypes, including pathogenic protein accumulation, cell-type-specific vulnerability, mitochondrial dysfunction, and abnormal neurotransmitter homeostasis^4^. We demonstrated that for the mDA neurons derived from patient-specific *PARK2*/*Parkin* and *PINK1* and mutant human iPSCs (referred to here as Parkin or PINK PD mDA neurons, respectively), the floor-plate based differentiation protocol displays differentiation properties comparable with those of control human iPSC or ESC (H9) lines (referred to here as WT mDA neurons). Both PINK1 and Parkin PD mDA neurons however, show increased levels of α-synuclein expression at the gene and protein levels. The PD iPSC-derived mDA neurons also exhibit increased susceptibility to mitochondrial toxins. Furthermore, we found mitochondrial abnormalities and increased intracellular dopamine levels in floor-plate-derived PD iPSC mDA neurons^4^. Consequently, all neurons we use here were produced using this directed differentiation protocol. Differentiation efficiency was assessed by the expression of the transcription factors FOXA2 and LMX1A that were expressed in greater than 80% of total cells for each of the WT and PD-hPSC lines tested^4^. Expression of tyrosine hydroxylase (TH), the rate-limiting enzyme for the production of dopamine was observed in greater than 50%, and nearly all cells (>90%) expressed the neuronal marker TUJ1 (Supplementary Fig. 2 and ref. ^4^).

**Fig. 1:**
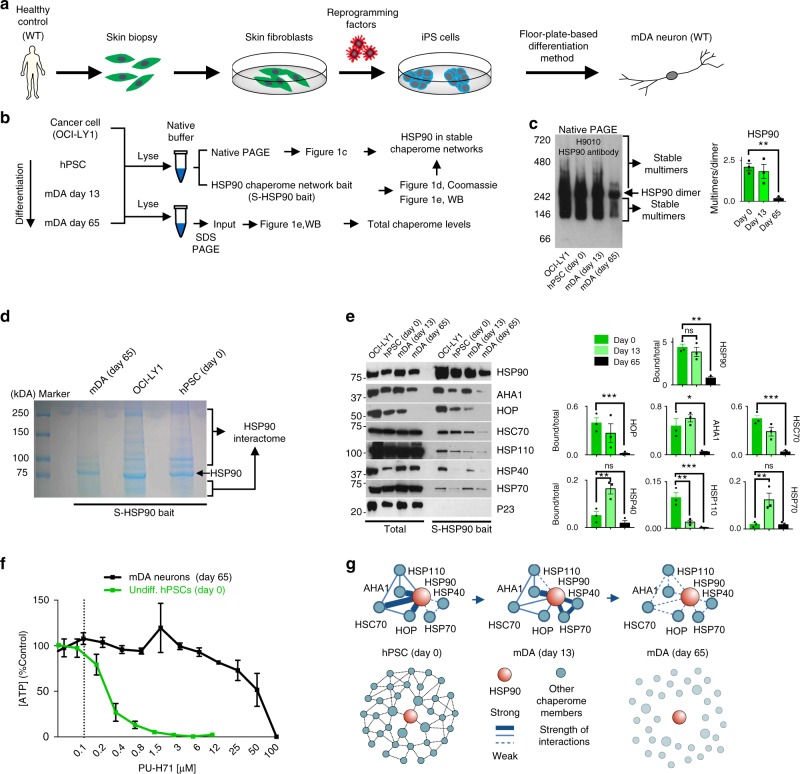
HSP90 complexes in hPSCs and hPSC-derived mDA neurons. **a**, **b** Schematic illustration of the overall experimental design, showing pluripotent stem cells (PSCs) differentiation into midbrain dopaminergic (mDA) neurons (**a**) and the methods used to determine HSP90 incorporation into stable chaperome networks (**b**). **c**–**e** Native-PAGE (**c**), Coomassie stained denaturing gel (**d**) and western blots (**e**) comparing chaperome member levels in the whole cell lysate (Total) with those in S-HSP90 complexes (either affinity-purified, (**e**) or retained under native conditions (**c**)) in: OCI-LY1 cancer cells, hPSCs (Day 0), hPSC-derived Day 13 precursors and hPSC-derived Day 65 mDA neurons. p23, pull-down specificity control. **c**, **e** Mean ± SEM, *n* = 3 individual values from the different experiments shown as points, One-way ANOVA with Dunnett’s post-hoc, ***p < 0.001; **p < 0.01; *p < 0.05; ns *p* > 0.05. **f** Viability of day 0 (hPSCs) versus day 65 mDA neurons in response to PU-H71 over 72 h. Dashed line, IC_50_ for OCI-LY1 is shown for reference. Graph, means ± SEM of data from three or four independent experimental replications. **g** Summary schematic, showing the disassembly of stable HSP90 networks, characterized by enhanced interaction between HSP90 and participant chaperomes, as the process of neuronal differentiation progresses from the PSC stage to the mature, day 65 DA neuron

To understand the role of stress on the neuronal proteome, we first assessed the biochemical nature of HSP90 during the differentiation of wild-type (WT) PSCs into mDA neurons (Fig. 1a)^3^. For cells at each differentiation stage, we used immunoblotting to compare total HSP90 and co-chaperone expression levels in whole cell lysate (Total) relative to levels of HSP90 integrated into the stable chaperome networks (S-HSP90 bait) (Fig. 1b). We have recently introduced two biochemical methods that can detect and quantify the incorporation of HSP90 in stable chaperome networks^27^. When applied to Native-PAGE, dynamic HSP90 complexes dissociate and HSP90 is seen as a dimer; however, when HSP90 is incorporated into complexes of enhanced stability with cochaperones and other cofactors, the basis of stable HSP90 chaperome networks, these complexes withstand separation and are detected upon immunoblotting^27^. The second method is based on the properties of PU-H71; the more HSP90 is incorporated into stable networks, the higher the affinity of PU-H71 for HSP90, and thus more S-HSP90 protein complexes (i.e., the S-HSP90 interactome) are captured on the bait^27^. The cancer cell line OCI-LY1 is used as a positive control for near maximal integration of HSP90 into stable chaperome networks^27^. We also include a pull-down specificity control—the co-chaperone p23—because the PU-H71 bait interacts specifically with HSP90 in a configuration that excludes p23 binding.

PSCs behaved similarly to the OCI-LY1 cancer cells (Fig. 1c–f). We found PSCs contained HSP90 incorporated into stable complexes with many interacting proteins, as demonstrated by their profile on Native-PAGE (Fig. 1c) and capture with the S-HSP90 bait (Fig. 1d, e). These S-HSP90 interacting proteins include co-chaperones previously shown^27^ to participate with HSP90 into the formation of epichaperome networks in cancer, such as HSP organizing protein (HOP, also known as stress-inducible phosphoprotein 1, STIP1), activator of HSP90 ATPase homolog 1 (AHA1, also known as AHSA1), HSC70 (heat shock cognate 70 kDa protein, the constitutively expressed HSP70 paralogue also known as HSPA8 and HSP7C) and its co-chaperone HSP110 (heat shock 105 kDa/110 kDa protein 1) (Fig. 1e). The similarity between PSCs and OCI-LY1 comes to no surprise and is supported by reports demonstrating that a MYC network accounts for similarities between embryonic stem and cancer cell transcription programs^35^. As reported in OCI-LY1^27^, and because the MYC-induced chaperome networks are required for cell survival, their dismantling by PU-H71 resulted in PSC cell death (Fig. 1f). During differentiation (13 days of differentiation) we also observed a remodeling of the HSP90 networks, where chaperones usually associated with acute stress, such as the inducible HSP70 and HSP40, showed an enhanced association with HSP90 (Fig. 1e).

Differentiation of PSCs (0 days of differentiation) into mDA neurons (65 days of differentiation) disassembled most of the stable HSP90 chaperome networks (Fig. 1c–f). While we observed comparable overall HSP90 in cells, a marked and gradual decrease in the levels of HSP90 engaged in chaperome network formation was evident. This was reflected in the disassembly of the HSP90 complexes under Native PAGE (Fig. 1c), diminished interactome (Fig. 1d) and co-chaperone presence in the PU-H71 bait (Fig. 1e), and a largely refractory response profile to PU-H71, where high concentrations of PU-H71 (≤50 µM over 72 h) showed minimal toxicity in day 65 mDA neurons (Fig. 1f). This profile is identical to that observed for non-malignant cells, both primary and cultured^27^. Thus, the process of differentiation was associated with stable chaperome network disassembly and the entry of the neuron into a state we associate with normal cellular proteostasis (Fig. 1g).

### PD-relevant stresses induce epichaperome networks

**Fig. 2:**
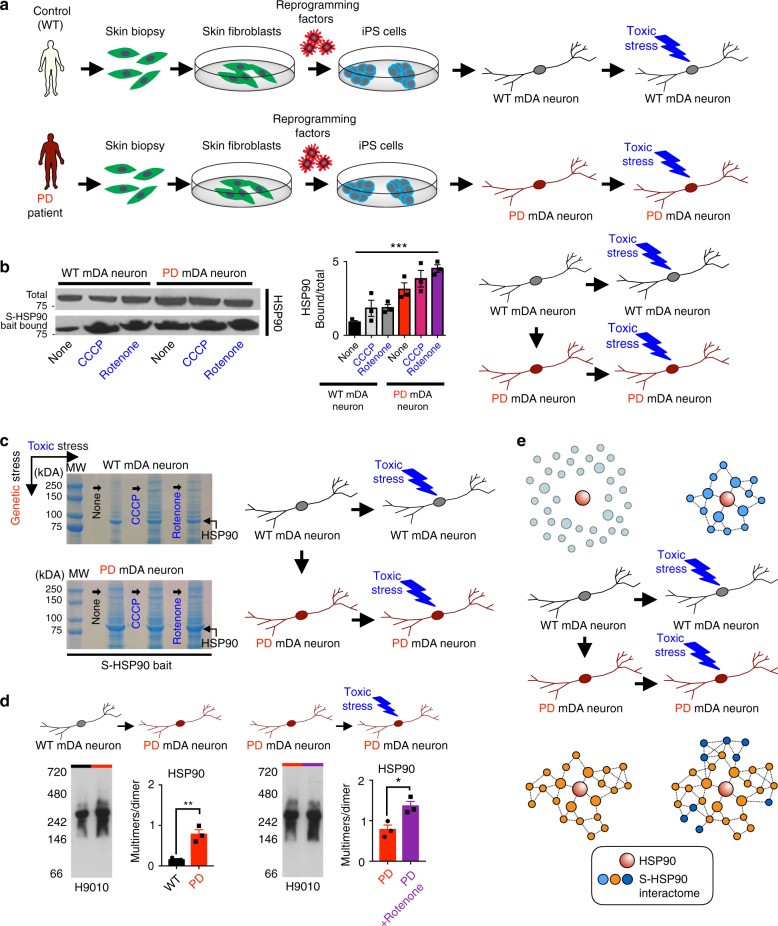
PD-relevant stresses reconfigure the chaperome into stable chaperome networks. **a** Schematic illustration of the overall experimental design showing the application of toxic and genetic PD stresses. **b** Western blot of total HSP90 and of S-HSP90 in mDA neurons upon genetic (*Parkin* defect) and toxin treatments (10 µM CCCP; 20 nM rotenone), alone or combined. Mean ± SEM, *n* = 3 individual values from the different experiments shown as points, One-way ANOVA, ***p < 0.001. **c** Coomassie stained denaturing gel (example of a gel submitted for proteomics analyses, *n* = 3 WT, *n* = 2 WT + CCCP, *n* = 2 WT + rotenone, *n* = 3 PD, *n* = 3 PD + CCCP, *n* = 3 PD + rotenone, where each *n* is a different experiment) showing the S-HSP90 and its interactome, as affinity-purified by the PU-H71 bait. **d** Native-PAGE shows S-HSP90 formation under PD stress, as indicated by an increase in stable HSP90 species over the dynamic HSP90 dimer. Mean ± SEM, *n* = 3 individual values from the different experiments shown as points, *t*-test, **p < 0.01; *p < 0.05. **e** Summary schematic showing how in mDA neurons genetic and toxic stresses induce chaperome network assembly, executed by an increase in the association strength between the participant proteins

We next tested whether disease-associated toxic stress or genetic stress conditions may trigger increased formation of HSP90-associated chaperome networks in mDA neurons (Fig. 2a, b). Mitochondrial dysfunction is an important contributor to PD pathogenesis^36^ and mitochondrial toxins such as the uncoupler CCCP have been previously used to model Parkin and PINK1-dependent defects in mitophagy^6,7^. Rotenone is a well-known pesticide that impairs mitochondrial function and is associated with PD risk^37^. As mentioned above, we previously assayed for and demonstrated the PD-related phenotypes in *Parkin* and *PINK1* iPSC-derived mDA neurons (mDA neurons derived from Parkin^V324A^ (Parkin) and PINK1^Q456X^ (PINK) mutant PSCs)^4^.

Here we found that at day 65 of differentiation, *Parkin*- and *PINK1*-mutant mDA neurons displayed high vulnerability to CCCP and rotenone, as measured by total ATP levels, which is a surrogate marker of cell viability (Supplementary Fig. 2a) and a reliance on oxidative phosphorylation as measured by use of the Seahorse platform to determine real-time oxygen consumption rates (Supplementary Fig. 2b). Neurons rely on oxidative phosphorylation to meet energy demands, and malfunctions of mitochondrial oxidative phosphorylation, such as induced by chronic exposure of the brain to the lipophilic pesticide rotenone, causes dopaminergic neuron degeneration^38^. Based on these observations, we proceeded to use both mitochondrial (induced by rotenone and CCCP, i.e., toxic stress) and genetic (induced by *Parkin* mutation, i.e., genetic stress, referred to as PD mDA neurons from here on) stress to elicit phenotypes and to understand how these stresses affect the mDA neuronal chaperome and the proteome in our cells (Fig. 2a). We first titrated in CCCP and Rotenone to identify a concentration window where neuronal stress rather than cell killing was the prevalent observed phenotype. To exclude gross clearance of mitochondria from neurons, which may result following CCCP and rotenone exposure, we performed quality control analyses on mitochondrial proteins^39^. We also used immunocytochemistry of dopaminergic markers NURR1 and TH to test for dopaminergic cell integrity upon such stress conditions (Supplementary Fig. 2a, 3 and ref. ^3^).

We observed that each of these PD-related toxic and genetic stresses augmented the number of proteins interacting with and forming stable complexes with HSP90 (Fig. 2b–d, Supplementary Fig. 3b, c and Supplementary Fig. 4). This was evidenced by the increase in S-HSP90 levels (Fig. 2b), in proteins integrated into the S-HSP90 networks visualized by Coomassie blue staining (Fig. 2c) and in the number of stable HSP90 complexes on Native PAGE (Fig. 2d). In addition to cell homogenates, we also validated in live cells that these stresses increased HSP90 participation in stable chaperome networks (Supplementary Fig. 4). For example, using PU-H71 linked to a FITC-fluorophore or a click-chemistry conjugate as a live-cell sensor^25,34,40^, we observed that toxin-treated cells showed significantly higher levels of fluorescent-PU-H71 signal, suggesting retention of the chemical sensor, and thereby increased incorporation of HSP90 into chaperome networks, in live cells^27^. Overall, these findings indicate that both genetic and toxic stresses induce stable chaperome network assembly, and that this remodeling of the chaperome network is executed by an increase in the association strength between the participant proteins (Fig. 2e).

### Chaperome network composition in PD-relevant stresses

**Fig. 3:**
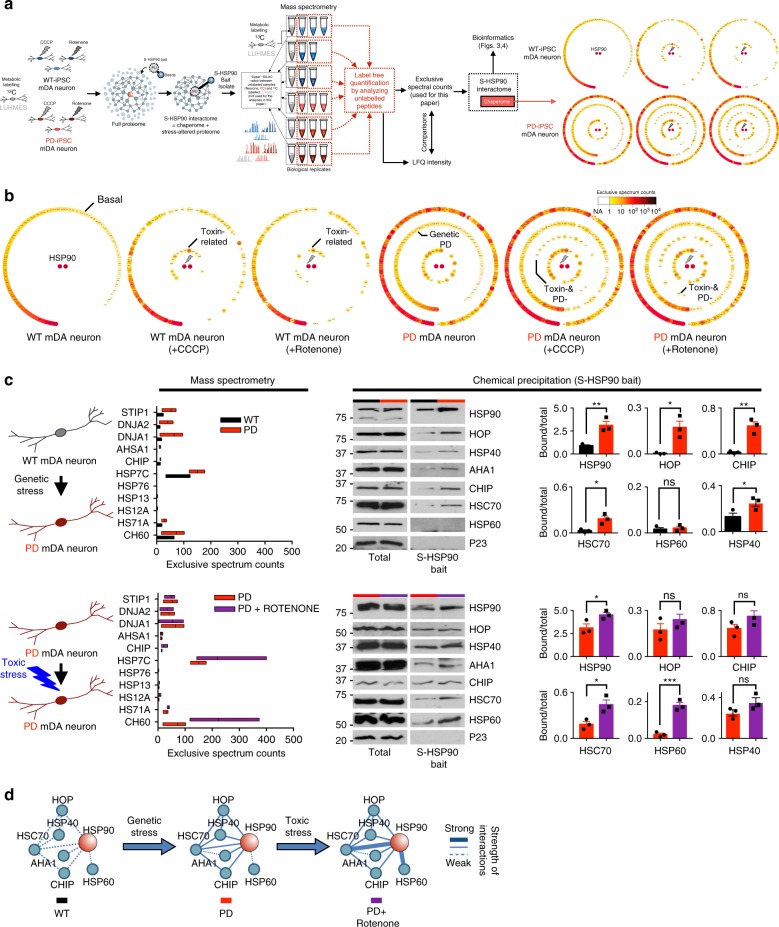
Chaperome network composition under PD-relevant stresses. **a** Schematic illustration of the experimental design for chemoproteomic studies and the identification of chaperome complexes. **b** Chaperome proteins associated with S-HSP90 in each stress condition. Large circles capture the chaperome protein classes identified in human PSC-derived 65-day mDA neurons for each condition. Small yellow to red colored circles represent known chaperome proteins and their expression levels from low to high. The outermost layer of small circles represents the set of chaperome members found in unstressed WT neuron and the matched set under the various stress conditions. The three inner layers of circles represent chaperome members enriched in PD (labeled genetic, outer circle), in CCCP and rotenone (10 µM CCCP; 20 nM rotenone; labeled toxin, middle circle) and the combination of genetic and toxic (inner circle). This is a vector graphic that can be enlarged for protein identity. **c** Mass spectrometry and western blot confirmation of S-HSP90 chaperome members under the indicated stress conditions. MS, Floating bar plot, lines inside the bars are mean; the range of the boxes corresponds to 25 and 75 percentile, *n* = 3 WT, *n* = 3 PD, *n* = 3 PD + rotenone, where each *n* is a different experiment. WB, Mean ± SEM, *n* = 3 individual values from the different experiments shown as points, *t*-test, ***p < 0.001; **p < 0.01; *p < 0.05; ns *p* > 0.05. **d** Summary schematic showing remodeling of the chaperome network under the indicated PD stresses

To characterize the composition of the chaperome networks under toxin or PD-genetic stress, we performed an unbiased chemoproteomics approach which takes advantage of the solid support-immobilized PU-H71 sensor (PU-H71 bait) to identify, capture, isolate and enrich in chaperome-bound HSP90 for a robust identification of its interactome by mass spectrometry^25,27^ (Fig. 3, see also Methods, Supplementary Data 1, 2 and Supplementary Figs. 5, 6). We performed a global analysis of HSP90-incorporating complexes in mDA neurons from control PSCs versus PD-PSCs (genetic stress), each under control conditions or following exposure to CCCP or rotenone (toxic stress, 10 µM CCCP or 20 nM rotenone) (Fig. 3a).

We first assessed stress-specific changes in the global composition of the HSP90-interacting chaperome (Fig. 3b) and validated a selected set by PU-bait chemical precipitation followed by western blot (Fig. 3c). Each PD stress was characterized by distinct global chaperome patterns depending on the specific genetic and toxic stress conditions (Fig. 3b). Chaperome network composition therefore may represent a stress-specific fingerprint for those mDA neurons. For example, HSP60 recruitment into the chaperome network was enhanced under toxic-stress (Fig. 3c), and also in certain cancer cells (Supplementary Fig. 7 and ref. ^27^). Competition with PU-H71 and HSP60/HSP90 immunoprecipitation experiments confirmed the specificity of HSP60 association with HSP90 complexes (Supplementary Fig. 7). HSP60 is a mitochondrial chaperone with relevance to PD; injury of mDA neurons results in increased HSP60 expression, which may create further damage to neighboring neurons after extracellular and microglia activation^41^. The functional consequences of this finding in the context of PD remains to be elucidated.

Under genetic stress, and to a lesser extent under toxic stress, we observed a significant increase in the participation of specific HSP70 machinery cofactors in the HSP90 chaperome networks (Fig. 3c). Among these are HOP and carboxy terminus of Hsp70-interacting protein (CHIP), both adaptors that link the HSP90 machinery to the HSP70 chaperone system^22,42^, as well as of HSC70 and its HSP40 family activators (the DnaJ heat shock protein family members DNJA1 and 2). These chaperome members were also found to participate in epichaperome network formation in cancer^27^, suggesting, overall, that a core chaperome was partly shared between genetic PD stresses and malignant stress in the formation of the stable chaperome networks. Collectively, these findings indicate that neuronal stress in PD may remodel the chaperome, in a stress-specific manner, and that in part, chaperome remodeling is executed by increasing the interaction strength between participating chaperome members and chaperome machineries (Fig. 3d).

### Proteome stress behind the chaperome networks in PD

**Fig. 4:**
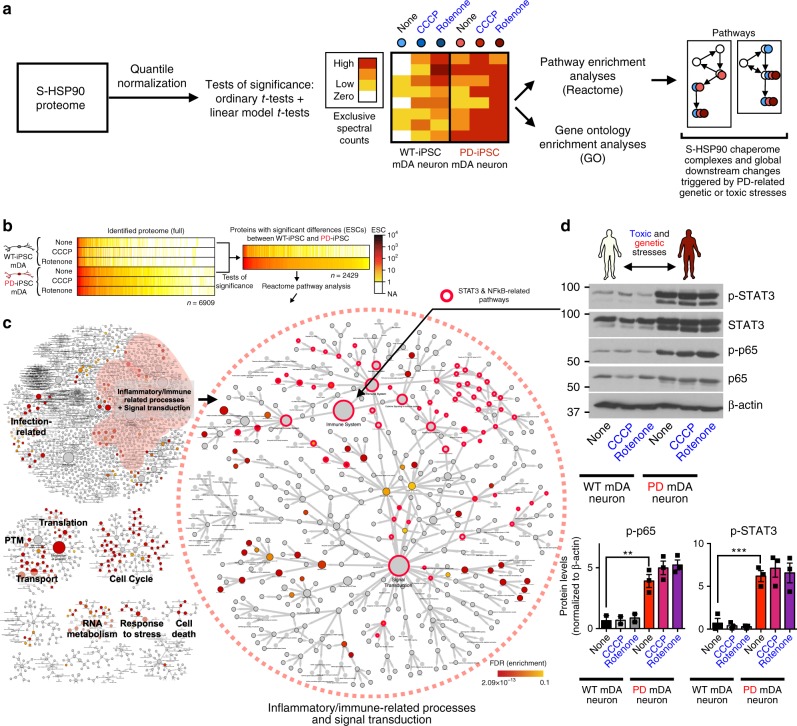
Global analysis of proteins and protein networks enriched in response to PD-relevant genetic and toxic stress in hPSC-derived mDA neurons. **a** Schematic illustration of the experimental design for chemoproteomic studies and the identification and validation of proteins and protein pathways enriched in S-HSP90 complexes under each PD stress. See Supplementary Data 1–4 for protein lists, statistical analyses and results of Reactome and GO enrichment analyses. **b** Heatmap of S-HSP90 bound proteins indicating those significantly enriched in mDA neurons under genetic (*Parkin* defect) stress. **c** Important cellular functions and pathways enriched under genetic stress. Inset shows the enrichment in inflammatory and signaling-related processes, with those incorporating STAT3 and NF-kB/p65-related pathways circled in red. (CCCP; 10 µM; rotenone; 20 nM). **d** Western blot confirms an increase in STAT3 and NF-kB/p65-activity in PD over WT mDA neurons, irrespective of the additional toxic stress. Mean ± SEM, *n* = 3 individual values from the different experiments shown as points, *t*-test, ***p < 0.001; **p < 0.01

Next, we investigated the broader protein networks associated with, and possibly buffered by the stress-induced remodeling of the chaperome networks (Fig. 4a, b). Similar to the chaperome composition, our chemoproteomics analysis showed broad, stress-specific and disease genotype-related changes in the composition of the proteome (Fig. 4b). Unbiased pathway enrichment investigations using Reactome (Fig. 4c, Supplementary Fig. 8a and Supplementary Data 3) and Gene Ontology (GO) analyses (Supplementary Fig. 8b and Supplementary Data 4) of the S-HSP90 interactome found signalosome/signal transduction and related inflammation/immune system processes, cell cycle, transcription, translation, metabolism, transport, cellular response to stress, and cell death with a large number of changes to be over-represented in a stress-dependent manner (Fisher’s exact test, FDR < 0.1, odds ratio > 1.5).

**Fig. 5:**
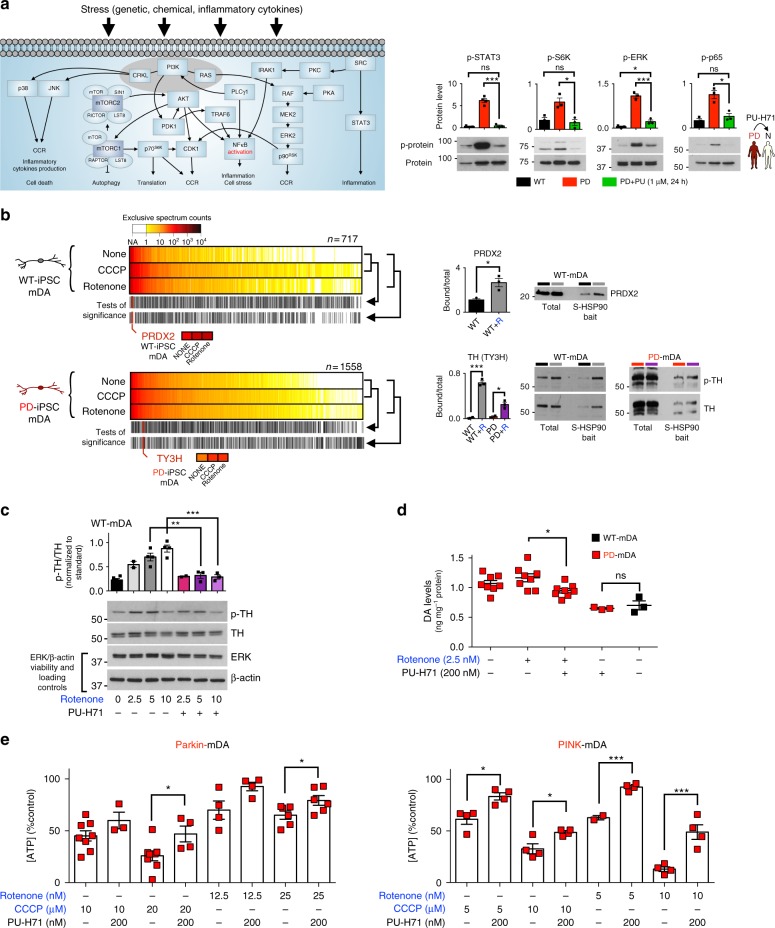
Validation of protein alterations and functional studies for pathways induced by genetic and toxic stress, and reversal of PD-related alterations following S-HSP90 inhibition. **a** Schematic illustration of key signaling proteins and pathways activated in response to genetic stress. Western blot confirms higher activity of these pathways in PD over WT mDA neurons, and shows that PU-H71 restores these to WT levels. Mean ± SEM, *n* = 3 individual values from the different experiments shown as points, *t*-test, ***p < 0.001; **p < 0.01; *p < 0.05; ns *p* > 0.05. **b** Heatmaps of proteins significantly enriched under toxic stress (10 µM CCCP; 20 nM rotenone) and their validation by western blot (Input, total levels and S-HSP90 bait isolate, chaperome network associated). Mean ± SEM, *n* = 3 individual values from the different experiments shown as points, *t*-test, ***p < 0.001; *p < 0.05. **c** Western blot and quantification of rotenone stress increase in the p-TH:TH ratio and its reduction by PU-H71 (200 nM). p-ERK/ERK, cell viability control; β-actin, protein loading control. Mean ± SEM, *n* = 3 individual values from the different experiments shown as points, One-way ANOVA with Tukey’s post-hoc, ***p < 0.001; **p < 0.01. **d** Total intracellular dopamine levels in PD mDA neurons in conditions of toxic stress and under PU-H71 rescue; Mean ± SEM, *n* *=* 4–5 individual values from the different experiments shown as points, *t*-test, *p < 0.05. **e** PU-H71 treatment of rotenone- or CCCP-stressed mDA neurons significantly increases their viability, as measured by total ATP levels; means ± SEM, *n* *=* 3–8 from independent differentiations, *t*-test, ***p < 0.001; **p < 0.01; *p < 0.05

Under genetic stress, we found an enrichment of pathways that lead to and are activated by inflammatory cytokines (Fig. 4b–d see STAT3- and NF-kB/p65-related pathways), suggesting that mDA neurons may be sensitive to changes related to inflammation or glia- and astrocyte-related responses, pathways that are strongly modulated by changes in STAT3 and NFκB signaling, and in turn, that neurons themselves may produce these neuroinflammatory cytokines, creating a self-destructive loop. Indeed, chronic neuroinflammation is one of the hallmarks of PD pathophysiology, and activation of glial cells and increases in pro-inflammatory factor levels are common features of the PD brain^43^. Inhibition of the JAK/STAT pathway was found recently to prevent degeneration of dopaminergic neurons induced by α-SYN-induced neuroinflammation by suppressing microglial activation, macrophage and CD4(+) T-cell infiltration and production of proinflammatory cytokines/chemokines^44^. Genetic stress was also associated with a significant increase of many signaling proteins with key nodal roles in modulating processes related to cell cycle re-entry (CCR), signaling, stress, translation, autophagy, and cell death, including members of JNK, PI3K/AKT/mTOR, MAPK/ERK, and STAT3 signaling pathways (Figs. 4c, d, 5a and Supplementary Data 2).

### Validation and functional analysis of molecular changes

We first validated candidate pathways that correlated with PD genetic stress (Fig. 4c, d). Immunoblot data confirmed that regardless of the specific toxin regimen, *Parkin* mutant mDA neurons showed increased levels of both total and phosphorylated forms of STAT3 and potentially of active NF-κB, as suggested by increased phosphorylated p65 levels (Figs. 4d, 5a and Supplementary Fig. 8c). As part of the NF-κB signaling pathway, p65 is typically involved in inflammatory and immune responses, and can be induced by several stressful stimuli. Aberrant NF-κB may be linked to defective synaptic plasticity but has also been linked to cancer^45,46^. Similarly, STAT3 also has key roles in inflammation and malignant transformation, where the persistent activation of STAT3 is found to mediate tumor-promoting inflammation, as it can promote pro-oncogenic and inflammatory pathways, including NF-κB and interleukin-stimulated JAK pathways^47^.

We also validated other nodal proteins identified in the genetic-stress altered neuronal proteome networks, such as of the PI3K/AKT/mTOR (see S6K activation) and MAPK pathways (see ERK activation) (Fig. 5a, Supplementary Fig. 8c). Dysregulation of the PI3K/AKT/mTOR pathway is commonly reported in brains from AD and PD patients, and linked to defective autophagy^48^. Autophagosomes are observed in postmortem brain tissue of PD patient, especially in the Lewy bodies, but our data suggest that processes leading to autophagy defects may start early in the disease. Similarly, chronic activation of ERK promotes neuronal cell death and is involved in a variety of mechanisms linked to triggering neurodegeneration; this is contrast to acute ERK activation which may be protective^49^. Only a subset of these pathway alterations, and in a less pronounced manner, were present in fibroblasts obtained from patients carrying *Parkin* mutations (homozygous c.1072delT, compound-heterozygous delEx3–4 + duplEx7–12; Supplementary Fig. 8d). While this observation may suggest a higher vulnerability of midbrain dopaminergic neurons to the effects of the evaluated genetic stresses, as previously proposed^50^, our sample size is too limited to draw a robust conclusion. Recent reports^4,51^ suggest that the vulnerability of mDA neurons may be attributed to dopamine itself.

Exposure to PU-H71, previously shown to interfere with stable chaperome network function^27^, restored p-STAT3, p-S6K, p-ERK and p-p65 to WT mDA neuron levels (Fig. 5a), confirming that these PD-pathogenic events are indeed S-HSP90 chaperome network propagated. STAT3 inhibition with the JAK inhibitor AZD1480 led to an immediate collapse of the stable chaperome complexes (as noted for HSP90 and HOP, see Supplementary Fig. 9) without inducing observable changes in the total levels of these chaperomes, suggesting an intricate interrelationship between the chaperome and the proteome in cellular stress conditions; this observation warrants future investigation and validation in mDA neurons across the various genetic and toxic stress conditions.

Toxin-stress conditions led to the association of TH, the protein which catalyzes the rate-limiting step of dopamine synthesis^52^, and PRDX2, an anti-oxidant protein, with the chaperome network formation (Fig. 5b). A pathogenetic role of TH has been suggested, as the enzyme is essential for producing dopamine and other reactive oxygen producing molecules and is also a target for radical-mediated oxidative injury. Dopamine may contribute to increased oxidative stress, mitochondrial dysfunction and cell death in mDA neurons during PD^53^. Western blot analysis confirmed that TH and PRDX2 levels were similar in lysates but under tight epichaperome control following stress (Fig. 5b and Supplementary Fig. 10). Treatment with rotenone led to a dose-dependent increase in the ratio of Ser40 phosphorylated (p-TH) to TH in mDA neurons, which could be partly rescued by PU-H71 (Fig. 5c). Given the important role of TH in dopamine production, we next asked whether toxin-induced stress could elicit higher levels of dopamine production in our cells. We observed an increase in intracellular dopamine levels in PD mDA neurons, which was significantly diminished by PU-H71 comparable to basal levels in the absence of toxin treatment (Fig. 5d).

Collectively, these data propose the stable chaperome networks as propagators of cellular proteome alterations induced by genetic and toxic stress, that in turn are detrimental to neuronal function. Indeed, and in addition to restoring proteome activity to that observed under normal cellular homeostasis (Fig. 5a, c, d), pharmacological epichaperome network inhibition also significantly increased the overall viability of mDA neurons under stress (Fig. 5e).

## Discussion

We here combine the power of human neurons differentiated from PSCs of normal and PD patients, and a chemical biology technique utilizing a biochemical sensor of proteins enriched during cell stress. Merged, the two systems culminate in a sensitive method to detect, in an unbiased manner, proteome-wide molecular alterations in mDA neurons that occur in response to stresses commonly used for modeling PD-related phenotypes. By pinpointing the nature of changes in proteome networks, pathways and/or in individual proteins, and by investigating alterations in protein–protein interactions upon stress, this method may therefore provide important information that is unavailable through, but complementary, to other omics methods.

In the past, one challenge in the neurodegenerative disease research field has been the limited availability of disease-relevant cells, such as primary neurons, to study disease-related signaling pathways. Most of the prior studies were based on heterologous cell systems using immortalized cell lines, or the use of genetic mouse models of the disease. However, both strategies have major drawbacks, such as the limited relevance of cell lines for studying neurodegeneration and the limited success in PD mouse models to recreate key disease-related phenotypes including the progressive loss of mDA neurons. The use of iPSC technology offers access to patient-specific cells, carrying the disease-causing genetic changes and to the specific neurons known to degenerate, at a scale suitable for biochemical studies. In the current study, we focused on providing proof-of-principle using *Parkin*-mutant PD iPSCs, as this genotype is known to affect mitochondrial homeostasis and function. We also included toxic mitochondrial stressors for their well-studied PD-related effects^4,54,55^. Follow-up studies should include additional PD-related genotypes to address whether the pathways identified here apply to PD-iPSC-derived mDA neurons across same and various genetic or even sporadic forms of the disease. An intriguing question that may be addressed with this method is the specificity of the insult, and how stressors such as progerin (aging stress) or α-synuclein may affect the chaperome and proteome networks in mDA neurons.

A limitation to using patient cells is that each patient genetic background is unique. PD results likely from the culmination of genetic and environmental risk factors. Therefore, without directly studying the gene of interest, it is difficult to discern the contribution of the patient’s genetic background and possible environmental exposure to the disease. One way to address this issue is the generation of gene-corrected lines. The use of CRISPR/Cas9 technology will make it possible to introduce or repair many such PD-related mutations within the same isogenic hPSC background. In theory, the resulting iPSC lines would be genetically matched except for the specific, disease-causing gene to be targeted. It is important to note, however, off-target effects during the gene correction process or clonal selection could still result in phenotypes that are not disease- or genotype-specific. Such cellular manipulations themselves are stresses which themselves may result in proteome changes detected by the chaperome, because, as we show, the chaperome will restructure after each stress, and each stress will induce a specific restructuring. An alternative strategy could be to assay more PD iPSC lines, and to confirm that the observed phenotypes are reproduced across many different control-versus-patient lines harboring the same mutations. In our current study, the main baseline is not a genetic background of a cell line but rather a normal chaperome- or proteostasis-background; we defined its characteristics for neurons in Fig. 1 (in addition to other cell types in ref. ^27^). To this baseline, we titrate in five stresses related to PD—these may have both genetic and environmental origins or a combination thereof. Accordingly, our study is focused on testing the impact of various stressors in an iso-proteostatic background (in analogy to isogenic).

The premise of this paper is based on chaperome biology in disease—the chaperome acts as a buffer to the myriad changes during stress and as a source of adaptation. We here show how we can take advantage of such relationship between proteome alterations and the chaperome, to detect via the latter (i.e., chaperome) the former (i.e., the proteome alterations). We detect these changes in neuronal models recently characterized to present Parkinson-related features (i.e., pathogenic protein accumulation, cell-type-specific vulnerability, mitochondrial dysfunction, and abnormal neurotransmitter homeostasis);^4^ and we use toxins associated with PD pathology, thus concluding that proteome alterations detected by the method are linked to PD. Further supporting this claim are the rescue experiments where HSP90-epichaperome network inhibition by PU-H71 reverted the altered protein networks to a WT mDA phenotype and rescued neuronal viability. Also supportive are analyses of PD brains postmortem that report on proteome hallmarks we here detect in the iPSC-derived neurons^21,43,48^, a result suggesting that these proteome alterations may start early in the disease process. It will be interesting in future studies to investigate whether cellular background such as neuronal subtype identity may distinctly influence chaperome and proteome rewiring, perhaps providing a clue to a higher vulnerability of certain neuronal populations to specific stresses.

We find that neuronal stress remodels the chaperome in a stress-specific manner and that in part, chaperome remodeling is executed by increasing the interaction strength between participating chaperome members, and between the chaperome and the proteome it regulates. These changes appear partly independent of overall chaperome levels, suggesting that increased connectivity, via an increase in interaction strength among chaperome members, is important during cellular alterations induced by neuronal stresses. Combined with previous studies^29,30^, an intricate chaperome remodeling, reflected by changes in both expression and interaction strength among members, appear to characterize neurodegeneration.

We report that protein networks regulated by the stable HSP90-chaperome networks include those associated with increased inflammatory activity, aberrant signaling and other molecular alterations damaging to neuronal function. We find several pathways altered by PD-related stresses are also associated with malignant stress^56^, suggesting a molecular commonality in the two diseases, yet driven by seemingly unrelated genetic or environmental factors. This stress-altered proteome, while perhaps beneficial during short term stress, could itself serve to propagate disease by over-correcting for protein activity in mDA neurons. For example, TH phosphorylation and its stabilization by S-HSP90 chaperome networks during acute stress could result in excess dopamine production. Excess dopamine production can be harmful to a cell, as dopamine is easily oxidized and can contribute to oxidative stress and a feed-forward mechanism of neurodegeneration. Also, chronic activation of aberrant signaling and of innate immune responses, including those mediated by microglia, can trigger neurotoxic pathways, mediating neuronal damage and leading to progressive degeneration in a number of neurodegenerative diseases^57^. We show here that S-HSP90 chaperome network inhibition can attenuate the over-activation of such PD-related pathways, and it remains to be investigated if altering the stable HSP90-chaperome networks via PU-H71 type compounds may be beneficial in in vivo PD models, and ultimately the human setting. Previous work has shown that treatment with PU-H71 rescues the axon growth retardation caused by overexpression of the LRRK2 G2019S mutation in neurons derived from LRRK2 G2019S transgenic mouse brains^15^, suggesting stable HSP90-chaperome networks as a more general mechanism used by neurons to regulate pathologic proteome stress in PD.

In addition to hereditary PD, *PARK2* genetic alterations are also common across human cancers, with the *PARK2* gene either mutated and/or deleted, and with copy number loss being the most frequent mode of alteration^58,59^. For example, *Parkin* defects are common in cancers associated with and driven by inflammatory components. In patients with lung cancer associated with chronic obstructive disease, loss of *PARK2* increases the expression of pro-inflammation factors and the activation of NFκB^60^. In osteosarcoma, Parkin is often downregulated, and this leads to activation of the JAK/STAT3 pathway^61^. In a variety of tumor types, Parkin dysregulation is associated with a loss of control of cell cycle components^58,62–64^. Thus, STAT3 and NFκB pathway dysregulation (among others), as we detected in our *Parkin* line using the chaperome sensor, may also be relevant in cancers that have in common a *Parkin* defect but are differentiated by many other variables such as genotype, proteome component, metabolomic signature, microenvironment, and age among others.

In conclusion, the use of chaperome networks and their chemical probes as biochemical sensors in hPSC-derived lineages may present a general strategy to identify early-event protein changes involved in neurodegenerative diseases. We detail how this method may yield the discovery of proteome and chaperome networks that can be disrupted to reverse PD phenotypes. Future efforts applied across many PD-related genotypes as well as sporadic disease could lead to the identification of molecular commonalities to classify, diagnose and treat PD. Furthermore, our study also suggests that given the lack of toxicity in mature mDA neurons, and their ability to suppress the detrimental activity of toxic and genetic stresses on mDA fitness, PU-H71 and related epichaperome network inhibitor compounds may represent a promising therapeutic avenue for PD.

## Methods

### Reagents

PU-H71 was synthesized as previously reported^65^. The PU-H71 bait and the fluorescently-labeled PU-H71 (PU-FITC) were generated as detailed in the supplementary methods section (see also refs. ^40,66^). Rotenone and CCCP were purchased from Sigma-Aldrich and AZD1480 from Selleckchem.

### Cell lines

The OCI-LY1 cell line was obtained from the Ontario Cancer Institute and was grown in Iscove’s Modified Dulbecco’s Medium (IMDM) containing 10% FBS and supplemented with penicillin/streptomycin. Cell lines BCP-1 (CRL-2294), NCI-H1975 (CRL-5908) and MDA-MB-468 (HTB-132) were obtained from ATCC and grown according to manufacturer’s instructions. Cells were authenticated using short tandem repeat profiling and tested for mycoplasma.

### Antibodies

Antibodies were obtained from commercial vendors and profiled for use in the system under study (assay and species). Antibodies (clones) have been validated by the suppliers. To ensure the specificity of the antibodies and consistency between different lots, newly purchased antibodies were tested using samples known to express the proteins to ensure consistency across lot numbers. All the antibodies and their characteristics are summarized in Supplementary Table 1.

### Culture of undifferentiated ESCs, iPSCs and fibroblasts

PD iPSCs generated by retroviral overexpression of OCT4, SOX2, KLF4, and C-MYC from patients with mutations in *PARK2*/*Parkin* (B125 line) or *PINK1* (L2122 line) were generously provided by the D. Krainc laboratory (Northwestern University Feinberg School of Medicine) and the Klein laboratory (University of Lübeck) and were previously reported^54,55,67^. ESC lines H9 (WA-09, passages 35–45) and iPSC lines B125–62 (passages 20–30; PARK2/PARKIN mutated line), L2122 (passages 20–30; PINK1 mutated line), and L2131 (passages 20–30; familial controls of PINK1 mutated patient) were maintained on mouse embryonic fibroblasts (Global Stem) in 20% knockout serum replacement (Invitrogen)-containing human ESC medium^68^ and passaged using Accutase (Innovative Cell Technology). All experiments described in this manuscript (unless otherwise indicated in Fig. 5e, Supplementary Figs. 2 and 8) were performed using the PD line B125–62 and the WT line L2131. This study had been reviewed by the Tri-SCI ESCRO Committee (protocol number 2010-09-001). Fibroblasts were obtained from the Klein laboratory^69^ and cultured in high glucose Dulbecco’s Modified Eagle’s Medium supplemented with 10% fetal bovine serum and 1% penicillin–streptomycin (all PAA, Pasching, Austria) at 37 °C, 5% CO_2_. All the lines used in this study and their characteristics are summarized in Supplementary Table 2.

### mDA neuron differentiation

A modified dual-SMAD inhibition protocol was used to direct cells towards floor plate-based mDA neurons^3,5^. At day 30 of differentiation, cells were replated on dishes pre-coated with polyornithine (PO; 15 µg mL^−1^)/ laminin (1 µg mL^−1^)/ fibronectin (2 µg mL^−1^) in Neurobasal/B27/L-glutamine-containing medium (NB/B27; Life Technologies) supplemented with 10 µM Y-27632 (until day 32) and with BDNF (brain-derived neurotrophic factor, 20 ng mL^−1^; R&D), ascorbic acid (AA; 0.2 mM, Sigma), GDNF (glial cell line derived neurotrophic factor, 20 ng mL^−1^; R&D), dibutyryl cAMP (0.5 mM; Sigma), TGFβ3 (transforming growth factor type β3, 1 ng mL^−1^; R&D), and DAPT (10 nM; Tocris). Two days after plating, cells were treated with 1 µg mL^−1^ mitomycin C (Tocris) for 1 h to kill any remaining proliferative contaminants. iPSC-derived mDA neurons were fed every 2 to 3 days and maintained without passaging until they were assayed at day 65. To prevent neurons from lifting off, laminin and fibronectin were supplemented into the media every 7–10 days.

### Notes on relevance of disease modeling in mDA neurons

In order to establish our model system, we obtained iPSCs reprogrammed from PD patients as well as healthy controls. First, we used genetic sequencing to confirm that following the reprogramming process and clone selection, the resulting PD patient iPSCs indeed still contained the respective homozygous mutations. We tested control and PD patient lines for each mutation and confirmed its specificity to the Parkin and PINK1 line. The (*PARK2*) c.1072delT was specific to the Parkin line, and the (*PINK1*) c.1366C>T was specific to the PINK1 line; these mutations were not apparent in the other PSC lines. We confirmed that our iPSC lines could be differentiated with a similar efficiency to human embryonic stem cells (hPSCs). We applied our highly efficient mDA neuron protocol and used it as a guide for the proper markers^3^ to differentiate and validate the proper developmental milestones of our PSC lines matured into mDA neurons. Dopaminergic cells exist in various parts of the brain, but the midbrain dopaminergic neurons originate from the floorplate. To assay for floorplate induction, we looked for co-expression of two transcription factors expressed in the floorplate, FOXA2/LMX1A. At day 13 of differentiation, all lines indeed displayed highly efficient induction at similar levels of these two markers. At day 30 and 45 of differentiation, we assayed for post-mitotic midbrain 42 dopaminergic markers, NURR1 and tyrosine hydroxylase (TH). Post-mitotic mDA neurons express NURR1, a transcription factor important for dopaminergic cell type specification and identity. At day 30 and 45, we confirmed the highly efficient generation of mDA neurons from hESCs and iPSCs. Further, cells expressed similar levels of post-mitotic dopaminergic markers, NURR1/TH and maintained floorplate expression as indicated by FOXA2 expression. We compared the results of our new protocol to that of the previous protocol, which goes through the rosette stage (“MS5 protocol”). The MS5-based protocol could also generate mDA neurons from hESCs and iPSCs. However, this protocol is less efficient and more complex because it relies on MS5 feeder cells. Most importantly, the dopaminergic cells that arise from this protocol likely do not originate from the floorplate because they have low floorplate marker expression, and therefore are not midbrain-specific. As expected, although PSCs could be differentiated at equal efficiencies, the MS5 protocol resulted in relatively low levels of FOXA2+, NURR1+ and TH+ cells. Low FOXA2 expression suggested that most cells did not go through a floorplate stage, and therefore the TH expression of most of the cells was not specific to the midbrain. In order to be able to use these mDA neurons for disease modeling, we needed to confirm that these cells could be maintained in culture as they mature so that they could be assayed in disease modeling studies. Therefore, we developed a modified mDA neuron differentiation protocol to extend the purity of the culture to at least 70 days. We added a passaging step at day 15 and another one at day 30, which helped to eliminate contaminating proliferating cells. Next, to eliminate remaining, contaminating progenitors, we treated cells with a DNA agent that kills proliferating cells (Mitomycin C). Marker expression of FOXA2+, TH+/NURR1+, TUJ1+ and Ki67− indicated floorplate origin, midbrain dopaminergic, neuronal and post-mitotic, respectively at day 70. We also confirmed that donor age prior to reprogramming (young donor = under 20 years, old = over 60 years) did not affect differentiation efficiency. These results gave us confidence that neither mutations in the iPSC lines nor their reprogramming affected the potential of the cells to be differentiated into mDA neurons for disease modeling^4^.

### Viability assays

At differentiation day 30, 100,000 cells were seeded onto 96-well plates (solid black plate; Corning). Two days later, cells were treated for 1 h with mitomycin C to eliminate remaining proliferative cells. Cells were fed on these plates until day 65, at which indicated treatments were added to the cells for 24–72 h. Assays were performed according to the manufacturer’s indications. CellTiter-Glo Luminescent Cell Viability Assay (Promega) which generates a luminescent signal proportional to the amount of ATP present in lysed cells, and LDH CytoTox 96® Non-Radioactive Cytotoxicity Assay, Promega, which generates a color proportional to the amount of lactate dehydrogenase (LDH) in lysed cells were used. All experiments were repeated at least three times and read for luminescence (CellTiter-Glo) or at 490 nm absorbance (CytoTox). Vehicle treated live cells were considered control, and percent control was calculated for CellTiter-Glo. For CytoTox, the ratio of LDH signal in the lysed cell divided by total LDH (released + lysed) was calculated. DAPI staining was used to visualize and quantify live cell nuclei morphology as another proxy for cell viability.

### Oxidative phosphorylation

Pluripotent stem cells or mDA neurons were seeded in their respective media at the indicated cell densities. XF Cell Mito Stress Test (Seahorse biosciences) assay was performed per manufacturer’s instructions. At day 30, the differentiated neurons are transferred to a Seahorse XF96 Cell Culture Microplate, to be analyzed on the Seahorse XF96 Extracellular Flux Analyzer. Preparation and assay conditions: the day prior, an Extracellular Flux Plate is filled with 200 μL of XF Calibrant Solution per well, and stored in a hypoxic incubator set to 37 °C. On day 65, the XF Mito Stress Test Assay Medium was prepared using 500 mL XF Base Medium, with 25 mM glucose, 0.227 mM sodium pyruvate, 5 mL GlutaMAX, and 2 mM glutamine. The media pH was set to 7.4, and is heated to 37 °C. Once heated, the existing media in the cell culture plate was aspirated, and 200 µL of the assay media was added per well. The plate was immediately stored in the hypoxic incubator for 60 min prior to running the assay. During this time, the following compounds were prepared for injections, using the assay media: 5 mL of 1.25 µM oligomycin (1st injection), 5 mL of 0.25 µM FCCP (2nd injection), and 5 mL of combined 1.25 µM antimycin and 1.25 µM rotenone (3rd injection). 20 µL of each compound was put into their corresponding injection ports for all the wells in the Extracellular Flux Plate. The run was started once the cell culture plate has been in the incubator for 30 min, starting off with inserting the Extracellular Flux Plate to calibrate the machine. After 60 min, the cell culture plate was inserted into the machine, and the assay was run using the standard operating procedure for the Mito Stress Test. After running the assay, the wells were normalized to protein content.

### Western blotting

Cells were collected in ice-cold phosphate-buffered saline without calcium or magnesium (PBS−/−) with a cell lifter (Corning, Tewksbury, MA). Cell pellets were rapidly frozen on dry ice and stored at −80 °C. Cell pellets were lysed in 50 mM Tris, pH 7.4, 140 mM NaCl and 1% NP40 lysis buffer containing leupeptin and aprotinin (Sigma Aldrich). Lysates were spun at 10,000 rpm spin for 20 min at 4 °C. Samples were diluted with 4X Laemmli sample buffer, boiled for 5 min at 95 °C, and loaded onto a NuPAGE 4–12% Bis-Tris precast gel (Life Technologies). Gel electrophoresis was performed at 200 V for 1 h. Gels were transferred to a nitrocellulose membrane, and probed with indicated antibodies in 5% Milk in TBS. HSP90β (SMC-107), GRP75 (SMC-133D) and HSP110 (SPC-195) antibodies were purchased from Stressmarq; HSP70 (SPA-810), HSC70 (SPA-815), HSP60 (SPA-806), HOP (SRA-1500), and HSP40 (SPA-400) from Enzo; HSP90α (ab2928), p23 (ab2814) and AHA1 (ab56721) from Abcam; CHIP (2080), S6K (2217), phospho-S6K (S235/236) (4858), S6 ribosomal protein (2217), phospho-S6 ribosomal protein (S235/236) (4858), COX IV (4850), Tom20 (42406), phospho-ERK (T202/Y204) (4377), ERK (4695), P65 (8242), phospho-P65 (3033), phospho-STAT3 (Y705) (9131), STAT3 (9139), phospho-TH (S40) (2791) from Cell Signaling Technology; TH from Novus (NB300–109); and β-actin (A1978) and PRDX2 (WH0007001M1) from Sigma-Aldrich. See also Supplementary Table 1.

### Immunocytochemical analyses

For staining, cell cultures were fixed in 4% paraformaldehyde. Species-specific Alexa dye conjugate secondary antibodies were used (molecular probes). Antibodies for immunofluorescence staining were the following: FOXA2 (SC-6554) from Santa Cruz Biotechnology; Lmx1a (AB10533) from Millipore; MAP2 (M1406) from Sigma-Aldrich; NURR-1 (PP-N1404–00) from Perseus Proteomics; tyrosine hydroxylase (P40101–150) from Pel-Freez; TUJ1 (MMS-435P) from Covance.

### Cell lysis for chemical precipitation or immunoprecipitation

Cells were lysed with Felts buffer (20 mM HEPES, 50 mM KCl, 5 mM MgCl_2_, 0.01% (w/v) NP-40, freshly prepared 20 mM Na_2_MoO_4_ (pH 7.2–7.3)) and added 1 µg µL^−1^protease inhibitors (leupeptin and aprotinin), using three successive freeze (in dry ice) and thaw steps. BCA kit (Pierce) was used according to the manufacturer’s instructions to determine total protein concentration.

### S-HSP90 detection in lysed cells

PU-H71-immobilized beads or control beads, containing an HSP90 inactive chemical (2-methoxyethylamine) conjugated to agarose beads, were washed five times in lysis buffer. Next, 80 µL bead conjugates were incubated at 4 °C with the indicated amounts of cell lysate (500 µg for analysis by western blotting, 1 mg for analysis by mass spectrometry), and the volume was adjusted to 500 µl with lysis buffer. Following incubation, bead conjugates were washed five times with the lysis buffer and proteins in the pull-down were run on a 4–12% gel and analyzed by western blotting or mass spectrometry.

### S-HSP90 detection in live cells

30 days old mDA neurons were plated into 24-well plates (Corning) at 500,000 cells per well and treated with mitomycin C two days later, and fed on these plates until they were treated with indicated drugs at day 65. FITC and Click assay followed by fluorescence microscopy were used to determine the amount of PU-H71 uptake in live cells as previously described^25^. For CLICK assay, 2 µM of PU-alkyne were used, and the fluorescence was elicited through a catalytic reaction as previously described^70^. See also Supplementary Methods.

### S-HSP90 detection by native gel

Protein lysates were loaded onto 4–10% gradient not containing SDS PAGE and were ran at 100 V for 4 h at 4 °C. Proteins were transferred to a nitrocellulose membrane in a transfer buffer containing 0.1% SDS for 1 h. Membranes were probed with the HSP90 or HOP antibodies, as indicated.

### Co-treatment experiments

65-days neurons were co-treated with 200 nM PU-H71 and increasing concentrations of rotenone or CCCP for 72 h. The cells were collected and processed for dopamine levels, viability or western blot, as indicated.

### Pharmacological inhibition of STAT3 activity

To analyze the effect of STAT3 activity on the reconfiguration of the stable chaperome complexes, MDA-MB-468 cells were treated with 3 µM of AZD1480 for the indicated times. Cells were then collected in native lysis buffer (20 mM Tris pH 7.4, 20 mM KCl, 5 mM MgCl2, 0.01% NP40), and were subjected for protein analysis. To detect the stable chaperome complexes, 10–20 μg of protein were loaded onto 4–10% native gradient gel and resolved at 4 °C using the protocol mentioned above. STAT3 activity and total levels of HSP90 and HOP were determined using the above described western blotting method.

### Preparation of samples and analysis of dopamine

Sample preparation: 65-day old mDA neurons were lysed manually in PBS containing 1% Triton X and 200 µM Ascorbic Acid (Sigma). After a 15 min-spin, the supernatant was collected. From each sample, an aliquot was removed directly to measure protein concentration in 2 technical replicates. The remaining amount was then divided into two technical replicates and immediately loaded for dopamine measurement.

Analysis of samples for dopamine: Concentrations of dopamine in samples were determined by high-performance LC-MS/MS. A standard curve was prepared fresh during each analysis. Compound analysis was performed on the 6410 LC-MS/MS system (Agilent Technologies) in multiple reactions monitoring (MRM) mode using positive-ion electrospray ionization. A Zorbax Eclipse XDB-C18 column (4.6 × 50 mm, 5 µm) was used for the LC separation, and the analyte was eluted under an isocratic condition (95% H_2_O + 0.1% HCOOH: 5% CH_3_CN) for 10 min at a flow rate of 0.4 mL min^−1^. For each sample, dopamine levels were standardized to protein levels and expressed as ng DA mg^−1^ protein.

### S-HSP90 chemoproteomics

We performed a global analysis of HSP90-incorporating complexes in mDA neurons from control PSCs versus PD-PSCs (genetic stress), each under control conditions or following exposure to CCCP or rotenone (toxic stress, 10 µM CCCP or 20 nM rotenone) (Fig. 3a). To ensure maximal capture of HSP90 complexes, especially when part of the normal chaperome networks, we performed these experiments under conditions of excess bait and by adding molybdate, a stabilizer of the dynamic HSP90 complexes characteristic under normal cellular proteostasis^27^. We performed initial pilot studies to include both label-free quantifications (LFQ), based on total peak intensities using MaxQuant and on spectral counting^71,72^, and quantitative proteome profiling using stable isotope labeling by amino acids in cell culture (SILAC)^73^. For SILAC we spiked in heavy-labeled LUHMES cells^74^ into each 65-day mDA neuron sample (Supplementary Fig. 5). LUHMES cells are immortalized fetal midbrain dopaminergic cells that can be expanded indefinitely and differentiated for 6 days. As opposed to 65-day iPSC-derived mDA neurons, LUHMES required much less time for SILAC incorporation because they can be expanded prior to a brief 6-day differentiation. We found that the label-free quantitation approaches, either by measuring and comparing the MS signal intensity of peptide precursor ions or by counting and comparing the number of matched MS2 spectra of a given protein, give good interactome coverage, and produce, overall, reproducible results among replicates (see Supplementary Fig. 6a and Supplementary Data 1). Moreover, we found that the interactomes derived from exclusive spectral counting (ESC) and the MaxQuant label-free quantification (LFQ) methods substantially overlap (Supplementary Fig. 6b and Supplementary Data 1). Because chemical isotopic labeling of many samples can be extremely expensive and time prohibitive and because the mix of labeled LUHMES may potentially interfere with the detection of the native interactomes, specific to either WT mDA or PD mDA neurons, we decided to proceed in further replicate analyses with the use of the label free methods. Nonetheless, we have deposited all the ESC, LFQ, and SILAC-derived interactomes for those interested in using them for future analyses (Supplementary Data 1).

### SILAC media and LUHMES cell lines

LUHMES cells were grown as previously described and harvested at day 6 of differentiation^74^ (Supplementary Fig. 5). SILAC Protein ID and Quantification Media were used (Thermo). L-Lysine-2HCl, 13C6 for SILAC and L-Arginine-HCl, 13C6 for SILAC (Thermo) were added to the “H” or Heavy labeled LUHMES cells. To determine SILAC incorporation, cells were passaged 1–4 times in above SILAC media prior to differentiation. Cell pellets were flash frozen at −80 °C, lysed and resolved in gel electrophoresis, and analyzed for H:L isotopic labeling to determine percent incorporation. For chemical precipitation, cells were lysed as described below, and lysates were mixed in a 1:1 ratio with 65-day old human pluripotent stem cell-derived mDA neurons.

### Sample preparation

Cell lysates were first pre-cleared by incubation with control beads overnight at 4 °C. Pre-cleared OCI-LY1 cancer cells, pluripotent cells or day 65 mDA neuron cell extract (1000 µg) in 200 µl Felts lysis buffer was incubated with PU-H71 beads (80 µL) for 4 h at 4 °C. Beads were washed five times with lysis buffer, proteins eluted by boiling in 2% SDS, and affinity purified protein complexes were resolved using SDS-polyacrylamide gel electrophoresis, followed by staining with colloidal, SimplyBlue Coomassie stain (Invitrogen Life Science Technologies, NY) and excision of the separated protein bands. The number of gel sections per lane averaged to be 14. Gel bands were completely destained with 50% methanol and 25 mM NH_4_HCO_3_ / 30% acetonitrile and diced into small pieces and dehydrated with acetonitrile and dried using vacuum centrifugation. The gel pieces were rehydrated with 12.5 ng mL^−1^ trypsin solution (Trypsin Gold, Mass Spectrometry Grade, Promega) in 50 mM NH_4_HCO_3_ and incubated at 37 °C overnight. Peptides were extracted twice with 5% formic acid / 50% acetonitrile followed by final extraction with acetonitrile. The tryptic peptides were desalted by using a 2 µL bed volume of Poros 50 R2 reversed-phase beads (Applied Biosystems) packed in Eppendorf gel-loading tips^75^. The purified peptides were diluted to 0.1% formic acid, and each gel section was analyzed separately by LC-MS/MS analysis with an Eksigent 2-D nanoHPLC coupled directly to an Orbitrap XL mass spectrometer (ThermoFisher Scientific) using our published protocols^76^. Additional analyses were performed using a Q Exactive mass spectrometer coupled to a Thermo Scientific EASY-nLC 1000 (Thermo Fisher Scientific, Waltham, MA) equipped with a self-packed 75 µm × 20 cm reverse phase column (Reprosil C18, 3 µm, Dr. Maisch GmbH, Germany) for peptide separation. The mass spectrometer was operated in data-dependent (DDA) mode with survey scans acquired at a resolution of 70,000 over a scan range of 300–2000 m z^−1^. Up to ten most abundant precursors from the survey scan were selected with an isolation window of 1.6Th and fragmented by higher-energy collisional dissociation with Normalized Collision Energies (NCE) of 27. The maximum ion injection time for the survey and MS/MS scans was 60 ms and the ion target value for both scan modes was set to 3e6.

### Mass spectrometry data acquisition

For exclusive spectrum counts (ESCs) analysis, all mass spectra were first converted to mgf peak list format using Proteome Discoverer 1.4 and the resulting mgf files searched against a human Uniprot protein database using Mascot (Matrix Science, London, UK; version 2.5.0; www.matrixscience.com). Decoy protein sequences with reversed sequence were added to the database to allow for the calculation of false discovery rates (FDR). The search parameters were as follows: (i) up to two missed tryptic cleavage sites were allowed; (ii) precursor ion mass tolerance = 10 ppm; (iii) fragment ion mass tolerance = 0.8 Da; and (iv) variable protein modifications were allowed for methionine oxidation, deamidation of asparagine and glutamines, cysteine acrylamide derivatization and protein N-terminal acetylation. MudPit scoring was typically applied using significance threshold score *p* < 0.01. Decoy database search was always activated and, in general, for merged LS-MS/MS analysis of a gel lane with *p* < 0.01, false discovery rate averaged around 1%. The Mascot search result was finally imported into Scaffold (Proteome Software, Inc., Portland, OR; version 4.7.3) to further analyze tandem mass spectrometry (MS/MS)-based protein and peptide identifications. X! Tandem (The GPM, thegpm.org; version CYCLONE (2010.12.01.1) was then performed and its results were merged with those from Mascot. The two search engine results were combined and displayed at 1% FDR. Protein and peptide probability was set at 95% with a minimum peptide requirement of 1. Protein identifications were expressed as Exclusive Spectrum Counts, ESCs, that identified each protein listed. In each of the Scaffold files that validate and import Mascot searched files, peptide matches, scoring information (Mascot, as well as X! Tandem search scores) for peptide and protein identifications, MS/MS spectra, protein views with sequence coverage and more, can be easily accessed. To read the Scaffold files, free viewer software can be found at http://www.proteomesoftware.com/products/free-viewer. The mass spectra files were also subjected to label-free quantitation (LFQ) as well as SILAC analysis, using MaxQuant^71^ proteomics data analysis workflow (version 1.6.0.1) with the Andromeda search engine^71^. Raw mass spectrometer files were used to extract peak lists which were searched with the Andromeda search engine against human proteome (Uniprot human fasta, 2014), and a file containing contaminants such as human keratins. Trypsin specificity with 2 missed cleavages with the minimum required peptide length was set to be seven amino acids. N-acetylation of protein N-termini, oxidation of methionines and deamidation of asparagine and glutamines were set as variable modifications. For SILAC analysis, labels were set to Lys6 and Arg6. For the initial main search, parent peptide masses were allowed mass deviation of 20 ppm. Peptide spectral matches and protein identifications were filtered using a target-decoy approach at a false discovery rate of 1%. Label-free quantification of proteins was activated to achieve a global proteome wide normalization of the intensities. The resulting MaxQuant data were imported into Scaffold for FDR calculations and SILAC ratio calculations. Scaffold Q + (version Scaffold_4.4.6, Proteome Software Inc., Portland, OR) was used to quantitate SILAC peptide and protein identifications. Peptide identifications were accepted if they could be established at greater than 5.0% probability to achieve an FDR less than 1% by the Scaffold Local FDR algorithm. Protein identifications were accepted if they could be established at greater than 29.0% probability to achieve an FDR less than 0.01% and contained at least 2 identified peptides. Protein probabilities were assigned by the Protein Prophet algorithm^77^. Proteins that contained similar peptides and could not be differentiated based on MS/MS analysis alone were grouped to satisfy the principles of parsimony. Acquired intensities in the experiment were globally normalized across all acquisition runs. Individual quantitative samples were normalized within each acquisition run. Intensities for each identified peptide were normalized within the assigned protein. The heavy labeled samples were placed into the reference channels that were in turn normalized to produce a 1:1 fold change. All normalization calculations were performed using medians to multiplicatively normalize data.

### Bioinformatics analyses and data representation

The exclusive spectrum count (ESC) values, an alternative for quantitative proteomic measurements, were used for cumulative representation of data (number of samples: WT = 3; WT + CCCP = 2; WT + Rotenone = 2; PD (PARKIN) = 3, PD with CCCP = 3; PD with Rotenone = 3). Figure 2c was generated using Cytoscape (3.5.1)^78^ based on a selected panel of chaperome proteins. Tests of significance: For enrichment analyses of proteins between samples (Figs. 3b, 5b, Supplementary Fig. 8a), statistics were performed using R (version 3.1.3) limma package. The data were transformed into logarithmic base 10, followed by quantile normalization within the same sample group (samples of WT-iPSC mDA and samples of PD-iPSC mDA) (Supplementary Fig. 6a). Proteins were excluded from analysis if only one replicate has a determined ESC value (potential contaminants), unless the ESC value is no less than 5. To select proteins with significant enrichment between groups, we performed tests of significance based on two statistical models: (1) one-sided Student’s *t*-test, generating a first set of *p*-values (t.pv) (2) linear models, which were fit to the preprocessed data and moderated standard errors were calculated using empirical Bayesian methods. The latter generated a moderated *t*-statistic that calculated a second set of *p*-values (limma.pv). *P*-value cutoffs for enriched proteins between WT-iPSC mDA (untreated) and PD-iPSC mDA (untreated) were set to t.pv < 0.1 and limma.pv < 0.25 with log (fold change) < 0. The *p*-value cutoffs for enriched proteins between PD-iPSC mDA (untreated) and PD-iPSC mDA (treated with CCCP or Rotenone) were set to t.pv < 0.25 and limma.pv < 0.25 with log (fold change) < 0. Enriched proteins were selected if they satisfied either the *p*-values cutoffs of the t.pv or limma.pv methods. Heatmaps were generated by R (version 3.1.3) with lattice package.

Reactome pathway enrichment analyses were performed using Reactome Pathway Database. The calculations of false positive rate (entities FDR) and enrichment ratio (entities ratio) were based on established method described in ReactomeWiki, section “Gene list Dataset”^79,80^. The Reactome pathway figure (Fig. 4b and Supplementary Fig. 8a) was generated in Cytoscape (v3.51)^78^. Each node represents a pathway including a collection of relevant proteins. Nodes are functionally interconnected in a hierarchical manner (high to low, indicated by arrowheads) and graphed as a tree structure. The significance of the enrichment (FDR) and the number of enriched proteins found in relevant pathways is reflected by a color gradient (red to blue), the size of the nodes and label. Irrelevant pathways in which no protein was related were represented as borderless nodes. Detailed information on the pathways presented in Fig. 4c is provided in Supplementary Data 3d. Detailed information on the pathways presented in Supplementary Figure 8a is provided in Supplementary Data 3e,f.

Gene ontology enrichment analysis (Supplementary Fig. 8b): the FDR (false discovery rate obtained by the Benjamini Hochberg procedure) and enrichment ratios (odd ratios) were calculated by Fisher’s exact test comparing each GO (biological processes) from the bait samples to that of the total 20203 documented and reviewed human proteins from the UniProt database^81,82^. Details on the 2 × 2 contingency table, schematic of the method, *p*-values and odd-ratios for each GO terms are included in Supplementary Data 4. The Treemap was generated in R using the package “treemap”. The R package “GSEAbase” was used to query GO slim terms. The full treemap keeps all GO terms that passed the cutoffs of FDR < 0.1 and odds ratio > 1.5. For treemaps, gradient colors represent the significance of the enrichment of GO terms (FDR). The size of the boxes represents the number of proteins. Labels with gray background represent the broader GO terms (GO slim) that comprise lower level GO terms.

Comparison of ESC and LFQ intensity for protein identification (WT iPSC mDA versus PD iPSC mDA) (Supplementary Fig. 6b): The LFQ intensity values were transformed into logarithmic base 10, followed by quantile normalization. Missing values were filled by minimal LFQ intensity value across samples. Enriched proteins between WT iPSC mDA versus PD iPSC mDA were determined by applying tests of significance as described above, with cutoffs set to t.pv < 0.05 and limma.pv < 0.05. The enriched proteins based on LFQ intensity were subjected to Reactome Pathway enrichment analyses as described above. The results were visualized in Cytoscape using the same color scale and node size as in the ESC set. Detailed information on the pathways presented in Supplementary Figure 6d, based on LFQ values, is provided in Supplementary Data 3g.

Quality control analyses on mitochondrial proteins: Mitochondrial proteins were selected if their corresponding GO cellular component terms contain the keywords: ‘mitochondrion’ or ‘mitochondrial’. Cytosolic proteins were selected if their corresponding GO cellular component terms contain the keyword ‘cytosol’ or ‘cytosolic’. For the bar-plots of protein counts, only proteins with average exclusive spectral counts >1 were taken into account. For the sum of untransformed LFQ intensity values, missing values were omitted in the calculations of mean for each group.

### Statistical analyses

We used Students *t*-test (to compare 2 groups) and ANOVA (to compare multiple groups). The raw data distribution approximated a normal distribution (Kolmogorov Smirnov normality test whenever appropriate number of replicates where available). Data are presented as mean ± SEM and were derived from at least 3 independent experiments. Data on replicates (*n*) is given in figure legends. Prism (version 6.0a; GraphPad) was used for data analysis and presentation. The experiments were not randomized. The investigators were not blinded to allocation during experiments and outcome assessment. Sample size was chosen based on our previous experience with these methods and analyses. No samples were excluded from analyses.

## Electronic supplementary material

Supplementary Information

Description of Additional Supplementary Files

Supplementary Data 1

Supplementary Data 2

Supplementary Data 3

Supplementary Data 4

## Data Availability

Primary data, such as raw mass spectrometry files, Mascot generic format files and proteomics data files created by Scaffold have been deposited in the MassIVE database (https://massive.ucsd.edu/ProteoSAFe/static/massive.jsp); MassIVE accession ID: MSV000082091. All other data generated or analyzed during this study are included in this published article and its supplementary information files and are available from the corresponding authors upon reasonable request.
