# Supplementary material for: HSP90-incorporating chaperome networks as biosensor for disease-related pathways in patient-specific midbrain dopamine neurons
Source: Nat Commun. 2018 Oct 19;9:4345. doi: 10.1038/s41467-018-06486-6 (PMC6195591; doi:10.1038/s41467-018-06486-6)
Supplement: Supplementary file 1 — Supplementary Information [file 41467_2018_6486_MOESM1_ESM.pdf]

Supplementary Information

HSP90-INCORPORATING CHAPEROME NETWORKS AS BIOSENSOR FOR DISEASE-RELATED PATHWAYS IN PATIENT-SPECIFIC MIDBRAIN DOPAMINE NEURONS

Kishinevsky S, Wang T, Rodina A, Chung SY, Xu C et al.

Content	Description	Page
**Cover Page**		**S1**
**Supplementary Fig. 1**	Design and use of the chaperome network biosensor for the identification of PD pathogenic events	**S2**
**Supplementary Fig. 2**	PD-neurons are susceptible to environmental toxins	**S3**
**Supplementary Fig. 3**	Experiments to identify and confirm mDA integrity under toxic treatment	**S4**
**Supplementary Fig. 4**	Validation of the biochemical sensor in live mDA neurons	**S5**
**Supplementary Fig. 5**	Validation and use of SILAC in LUHMES cells	**S6**
**Supplementary Fig. 6**	Proteomics datasets analyses	**S7**
**Supplementary Fig. 7**	Validation of HSP60 association with S-HSP90 complexes	**S8**
**Supplementary Fig. 8**	Protein networks and biological functions enriched under PD-related stress	**S9**
**Supplementary Fig. 9**	Interrelationship between the stress chaperome and proteome	**S10**
**Supplementary Fig. 10**	Tyrosine hydroxylase is regulated by the S-HSP90 networks specifically under PD-related stress	**S11**
**Supplementary Table 1**	Antibodies used in the study and their characteristics	**S12**
**Supplementary Table 2**	Lines used in this study and their characteristics	**S13**
**Supplementary Methods**	Synthesis and characterization of chemical biology tools	**S14**
**Supplementary References**		**S19**

Supplementary Figures

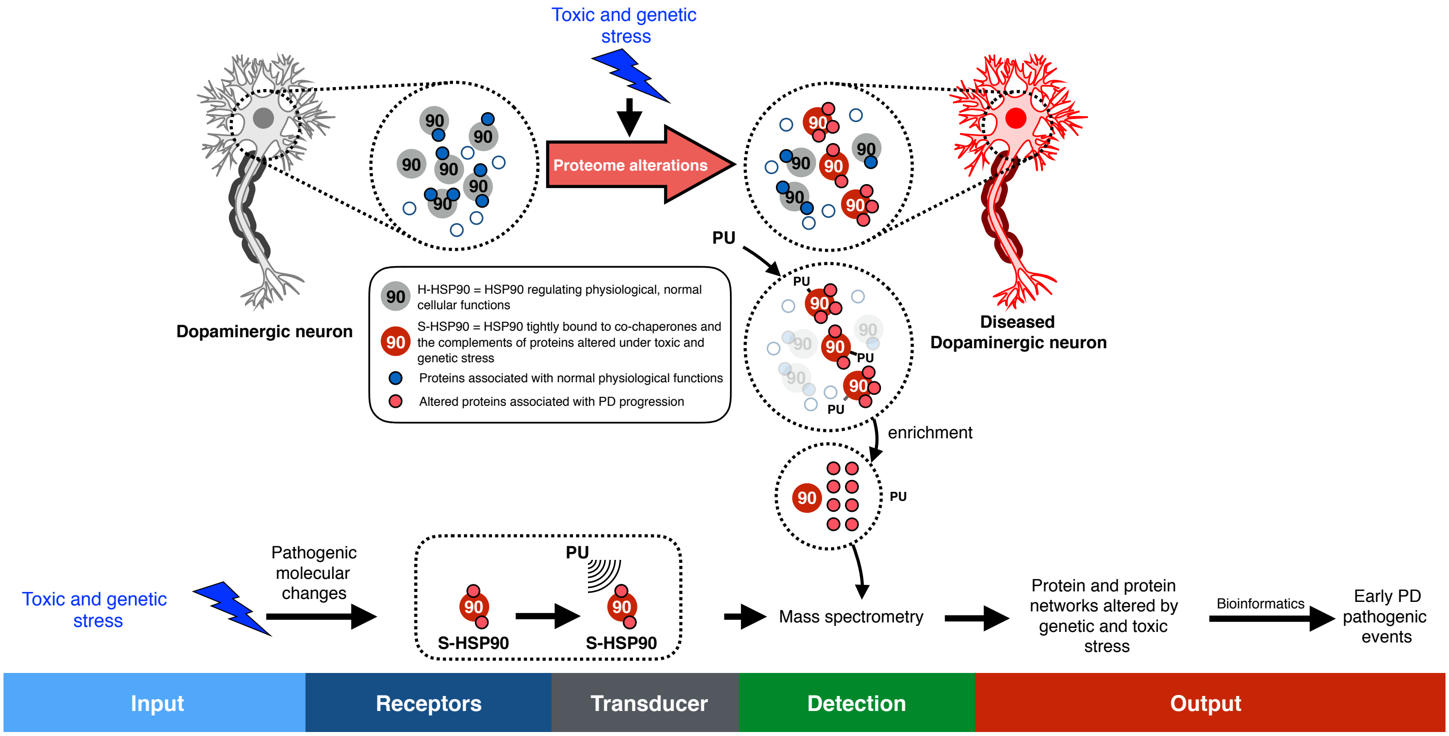

Supplementary Fig. 1. Design and use of the chaperome network biosensor for the identification of PD pathogenic events. Environmental and genetic risk factors contribute to PD pathogenesis and the associated loss of midbrain dopamine (mDA) neurons. The combination of human pluripotent stem cell (hPSC) technology and chemical probes for HSP90-incorporating chaperome networks results in a biosensor for identification of early PD pathogenic events. PD-related stresses (Input) are recognized by the S-HSP90 pool (Receptor) and in turn, these changes are detected and transformed into a measurable signal by PU-H71 (Transducer). The detected signal is measurable and quantifiable by a variety of methods, including mass spectrometry (MS) (Detection). Data resulting from MS are then analyzed by bioinformatics to yield protein and protein networks associated with PD pathogenesis (Output). Our method is based on the ability of a chemical probe to isolate the S-HSP90 chaperome network and its interactome, and consequently, it is limited to the analysis of the HSP90-associated chaperome networks. Certainly, other chaperome members and machineries, not in direct interaction with HSP90, may play an important role in PD. Future studies using systems-level multi-nodal polypharmacology, genetic reagents and related measurements will be needed to address the contribution of other chaperomes to formation of PD-relevant networks.

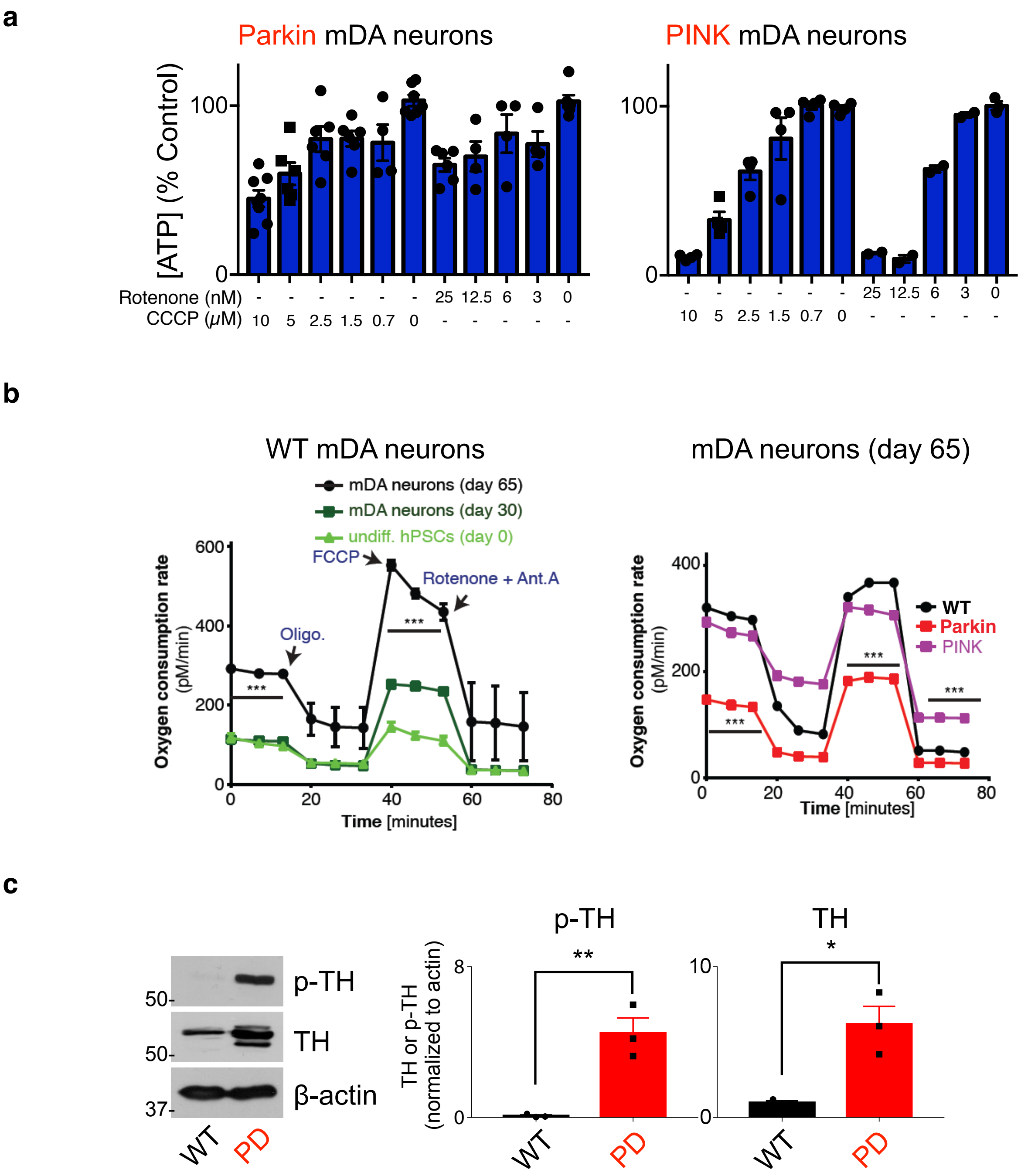

Supplementary Fig. 2. PD-neurons are susceptible to environmental toxins. **a** Viability of day 65 mDA neurons (means ± SEM, n = 3 to 8 from independent differentiations) in response to rotenone or CCCP over 72h. **b** Seahorse assay to measure oxygen consumption in WT undifferentiated hPSCs and hPSC-derived mDA neurons at day 30 and 65 of differentiation. Mean ± SEM; n = 3 independent differentiations of hPSCs; ANOVA; WT neurons vs PSCs; *** = p<0.001 and WT vs PD neurons; *** = p<0.001. **c** Western blot confirms p-TH and TH expression in the differentiated mDA neurons. Mean ± SEM, n = 3 individual values from the different experiments shown as points, t-test, ** = p<0.01; * = p<0.05.

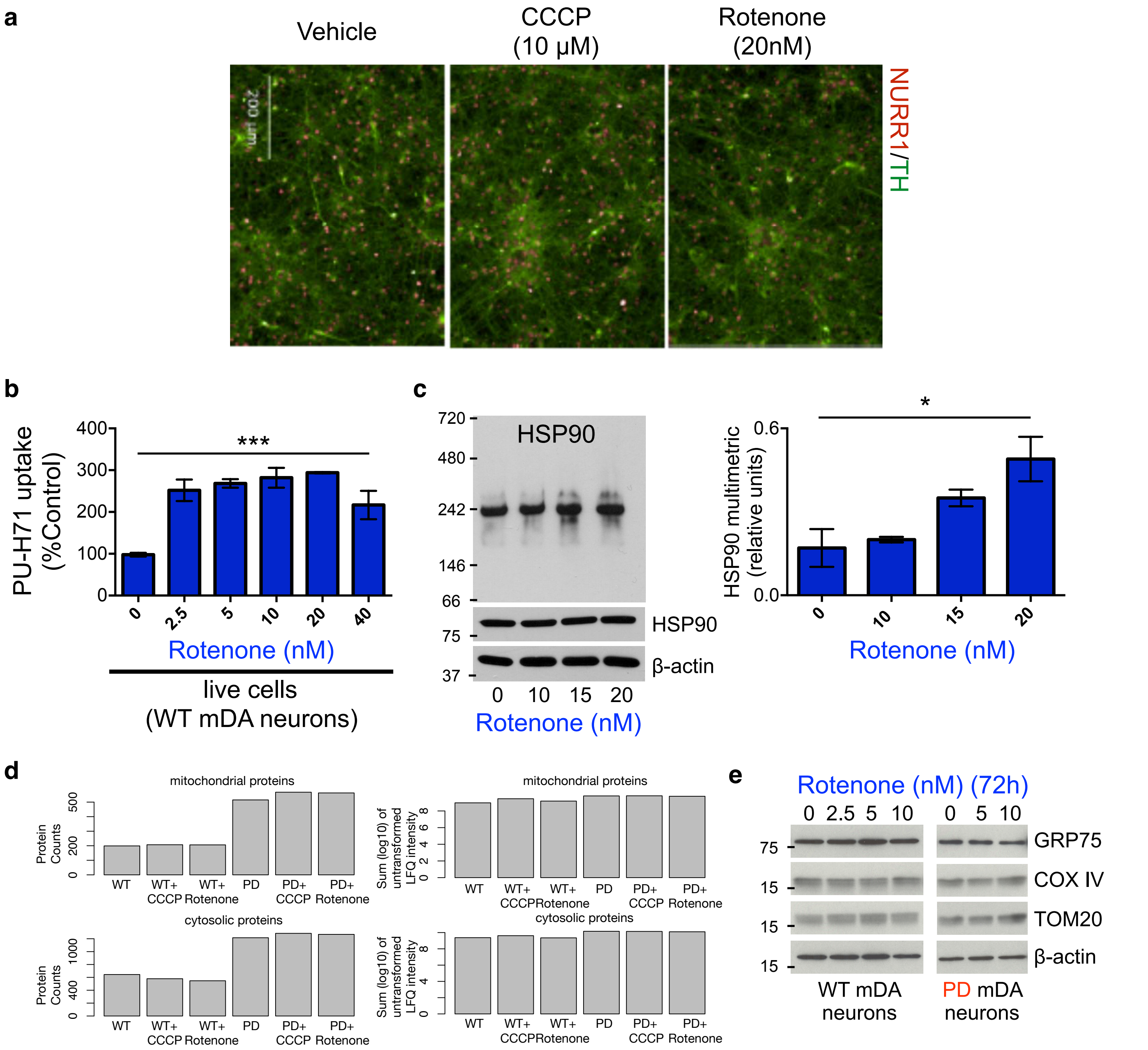

Supplementary Fig. 3. Experiments to identify and confirm mDA integrity under toxic treatment. **a** Representative immunocytochemistry of dopaminergic markers NURR1 (red), TH (green) confirms dopaminergic cell integrity upon stress conditions. Scale bar, 200µm. See also ref. 4. **b,c** Rotenone stress (72- and 24-h treatment, respectively) increases S-HSP90 levels as measured by fluorescent PU-H71 incorporation into live mDA neurons (**b**) and by Native PAGE analysis of HSP90 (**c**). Mean ± SEM, n = 3 individual values from the different experiments shown as points, One- way ANOVA, *** = p<0.001; * = p<0.05. **d** Proteins annotated to be either mitochondrial or cytosolic (for comparison) were pooled from Supplementary Data 1 (n = 3 WT, n = 2 WT+CCCP, n = 2 WT+rotenone, n = 3 PD, n = 3 PD+CCCP, n = 3 PD+rotenone, where each n is a different experiment). For each condition, the number of proteins and their LFQ intensity values (as a surrogate measurement of relative protein abundance among samples) were graphed to indicate no gross mitochondrial clearance upon the CCCP or rotenone treatments (10µM CCCP; 20nM rotenone). Western blot analyses for mitochondrial proteins confirm no gross changes in the steady-state levels of mitochondrial proteins. Western blot is representative of and was performed on two randomly selected sample sets from Fig. 5.

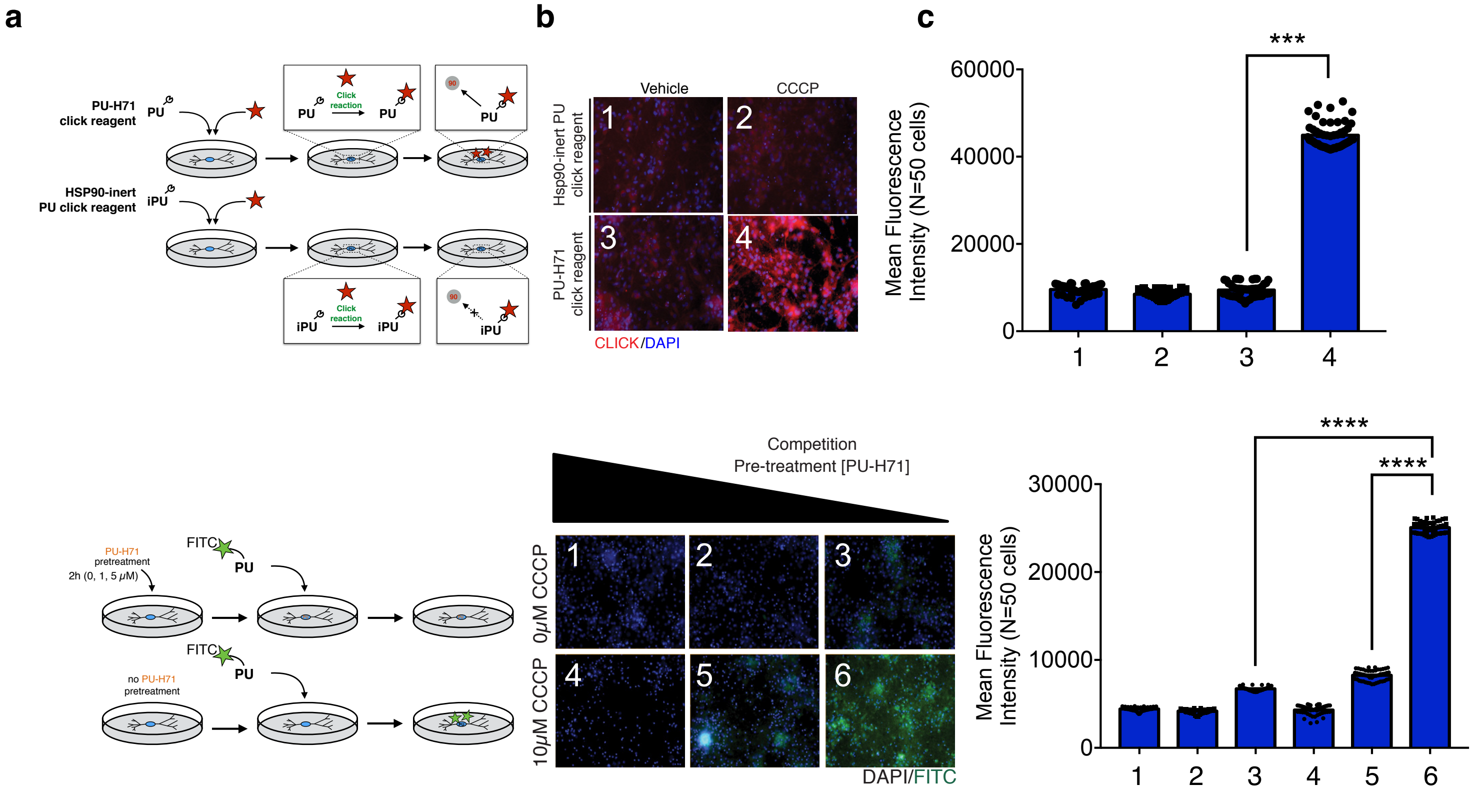

Supplementary Fig. 4. Validation of the biochemical sensor in live mDA neurons. **a** Schematic illustration for the use of fluorescently labeled PU-H71 to measure the levels of S-HSP90 (HSP90 participating in stable chaperome networks, i.e. epichaperomes) in WT mDA neurons exposed to CCCP. **b** Fluorescence microscopy analysis of cells treated with toxin (10μM CCCP) or vehicle for 72 h, then assayed with a PU-H71-probe, as indicated. To validate the specificity of the S-HSP90 sensor, toxin-stressed cells were pre-treated with PU-H71 for 2 h prior to assay with the PU-FITC probe, or were treated with a Control probe, as indicated. **c** Data were graphed as Mean ± SEM, n = 50 neurons where individual values from the different neurons are shown as points, t-test, **** = p<0.0001; *** = p<0.001.

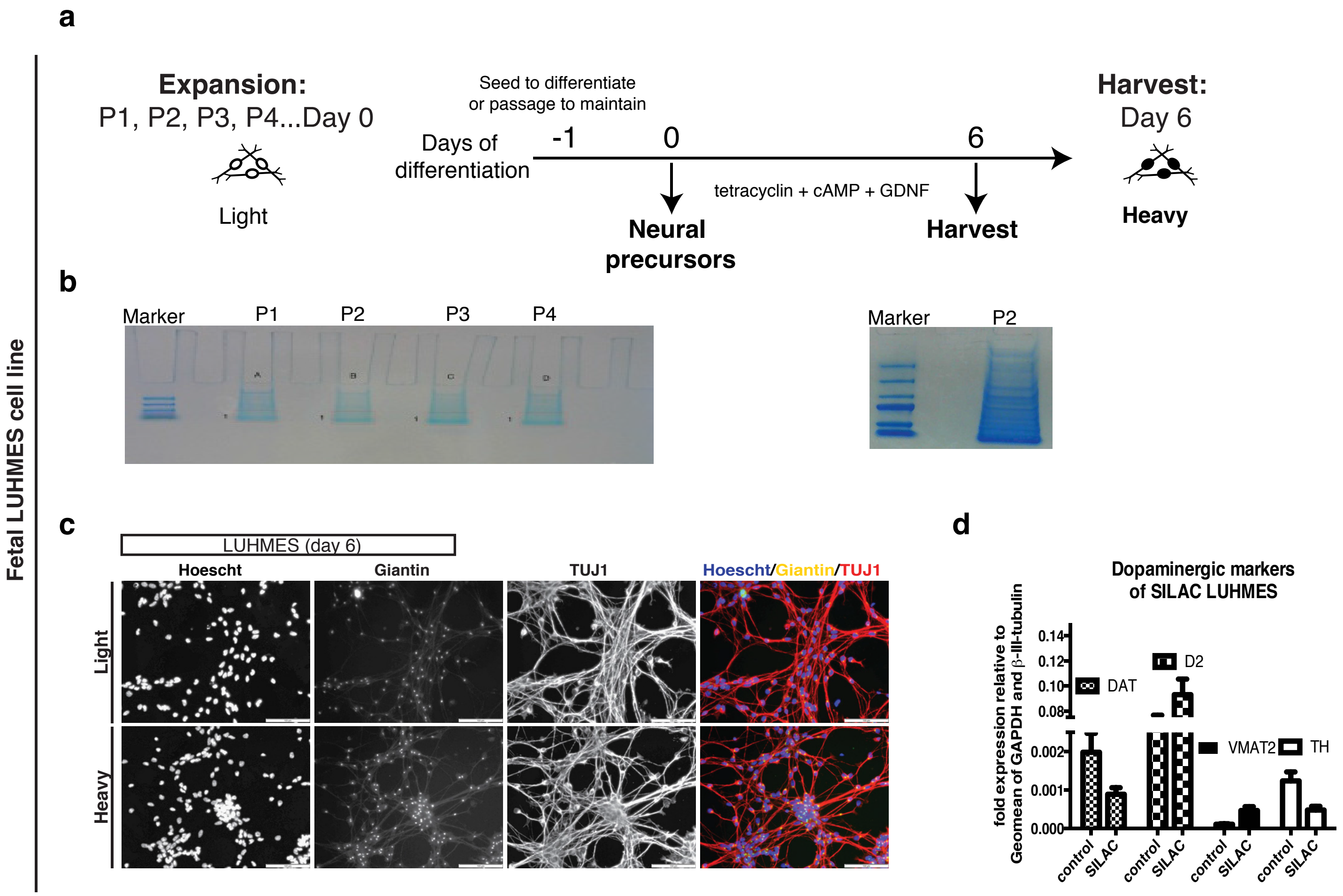

Supplementary Fig. 5. Validation and use of SILAC in LUHMES cells. **a** Schematic illustration of generating and labeling fetal-derived, immortalized LUHMES cells as a Heavy control for SILAC studies. During expansion phase, cells received Heavy labeled or Light (regular, unlabeled) media for one to four passages. Next, cells were differentiated for 6 days in Heavy or Light media. **b** Gels for analyzing percent incorporation in LUHMES lysate. At 2, 3 and 4 passages, Heavy media incorporated at 93%. Therefore, for mass spectrometry, cells were expanded for 2 passages + differentiated. **c** Heavy and Light LUHMES cells display comparable morphologies and neuronal marker expression by immunofluorescence. Giantin, Golgi protein. TUJ1, neuron specific tubulin marker. Hoechst, nuclear marker. **d** Heavy and Light labeled LUHMES cells express similar gene expression levels of dopaminergic markers: DAT, dopamine transporter (SLC6A3) D2, dopamine receptor D2 (D2R), VMAT2, vesicular monoamine transporter 2, TH, tyrosine hydroxylase. Control = Light, SILAC = Heavy labeled neurons (means ± SEM, n = 3 differentiations).

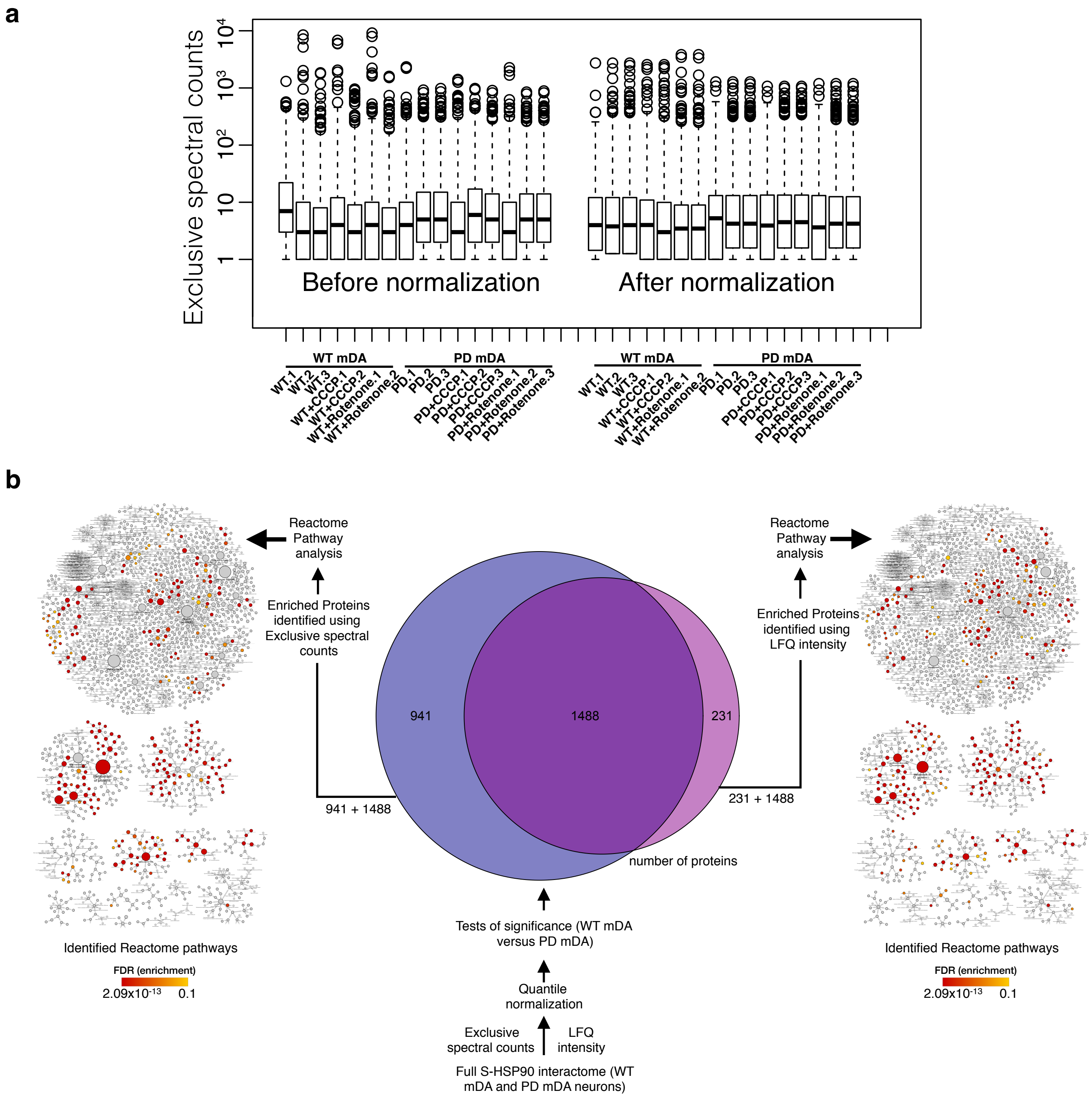

Supplementary Fig. 6. Proteomics datasets analyses. **a** Quantile normalization was applied to decrease intragroup variation between replicates of the test data (n = 3 WT, n = 2 WT+CCCP, n = 2 WT+Rotenone, n = 3 PD, n = 3 PD+CCCP, n = 3 PD+Rotenone, where each n is a different experiment). **b** Venn diagram and Reactome analyses show the overlap between the ESC and MaxQuant LFQ identified interactomes. See Supplementary Data 1 for full interactomes, Supplementary Data 2 for enrichment analyses over each stress experimental condition, and Supplementary Data 3 for Reactome analyses.

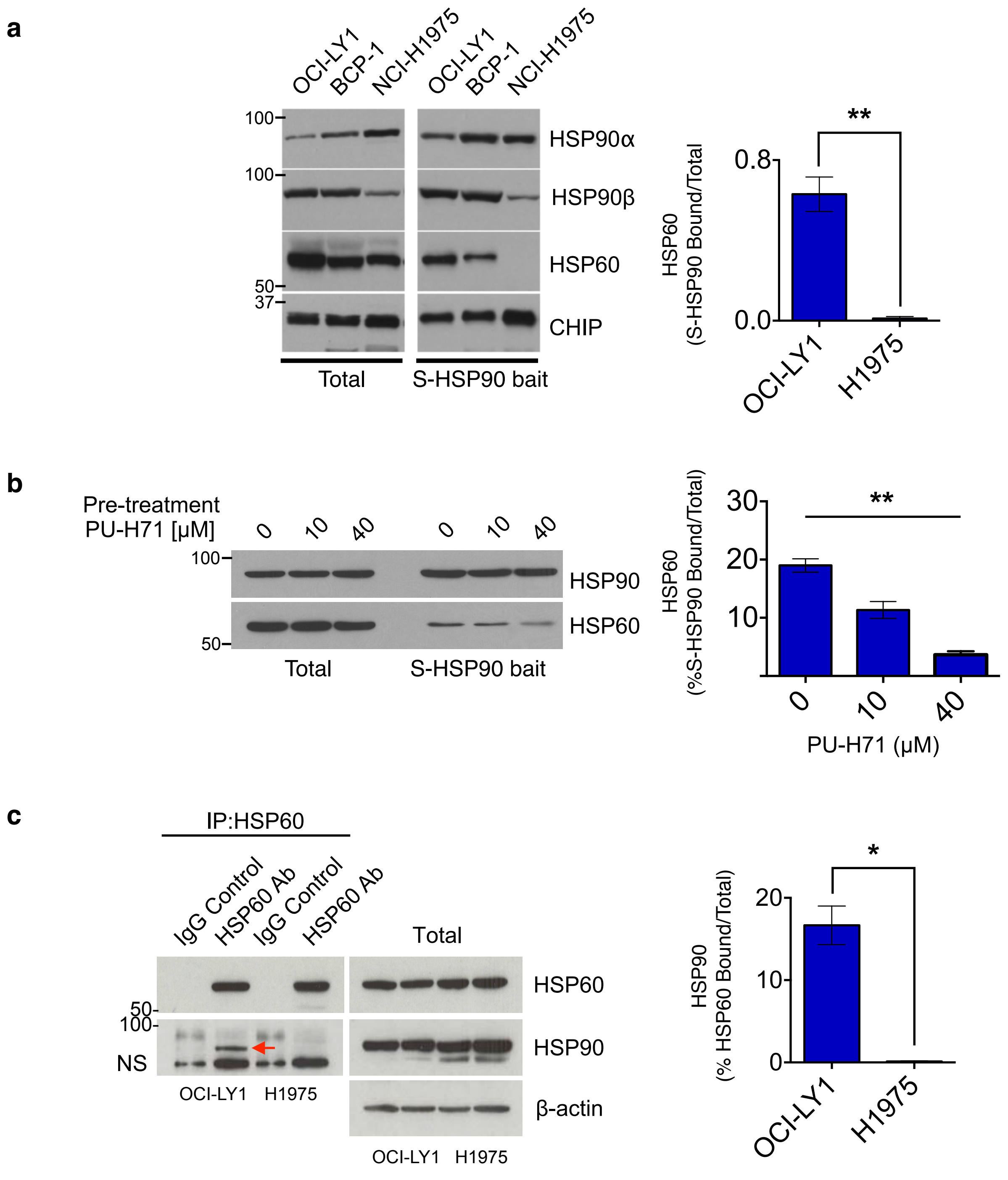

Supplementary Fig. 7. Validation of HSP60 association with S-HSP90 complexes. **a** Western blot of HSP90, HSP60 and CHIP in various cancer cell lines comparing total levels in lysate versus those in S-HSP90, as detected by affinity purification on the PU-H71 bait. Mean ± SEM, n = 3 individual values from the different experiments shown as points, t-test, ** = p<0.01. **b** Pre-incubation of OCI-LY1 lysates with PU-H71 followed by pull-down with the S-HSP90 bait results in less affinity capture of HSP90 and HSP60, suggesting specific interaction of HSP60 with the S-HSP90. Mean ± SEM, n = 3 individual values from the different experiments shown as points, t-test, ** = p<0.01. **c** Immunoprecipitation (IP) with an HSP60-specific antibody and an IgG control shows HSP60 interaction with HSP90 in OCI-LY1 but not in NCI-H1975 cells, confirming the results obtained with the S-HSP90 bait (see panel **a**). Total = lysate levels; NS, non-specific. Mean ± SEM, n = 3 individual values from the different experiments shown as points, t-test, * = p<0.05.

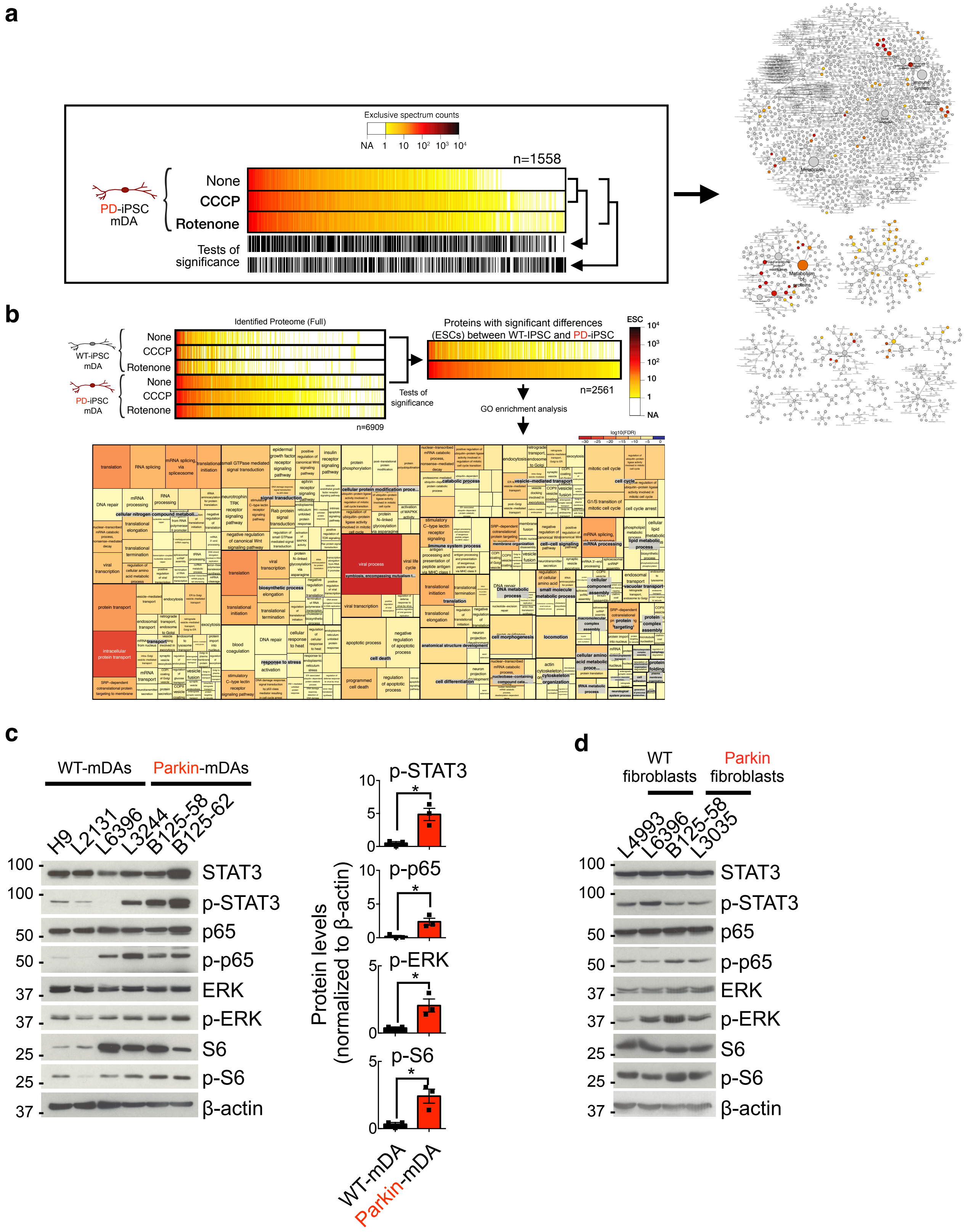

Supplementary Fig. 8. Protein networks and biological functions enriched under PD-related stress. **a** Heatmap and Reactome analyses show S-HSP90-regulated proteins and protein networks enriched by either CCCP or rotenone stress (10μM CCCP; 20nM rotenone) in mDA neurons. **b** Heatmap of proteins enriched under genetic stress and Gene Ontology (GO) enrichment analyses on the interactome dataset showing the treemap representation of most overrepresented GO terms (biological processes; FDR < 0.1 and enrichment ratio > 1.5). See Supplementary Data 4 for a full analysis. **c,d** Western blot analysis of STAT3, NFkB, ERK and S6K-pathway (measured by the activity of the downstream S6 protein) activity in PD and WT mDA neurons (**c**) and fibroblasts (**d**). Mean ± SEM of values from the three individual neuronal lines, each shown as points, t-test, * = p<0.05 (**c**).

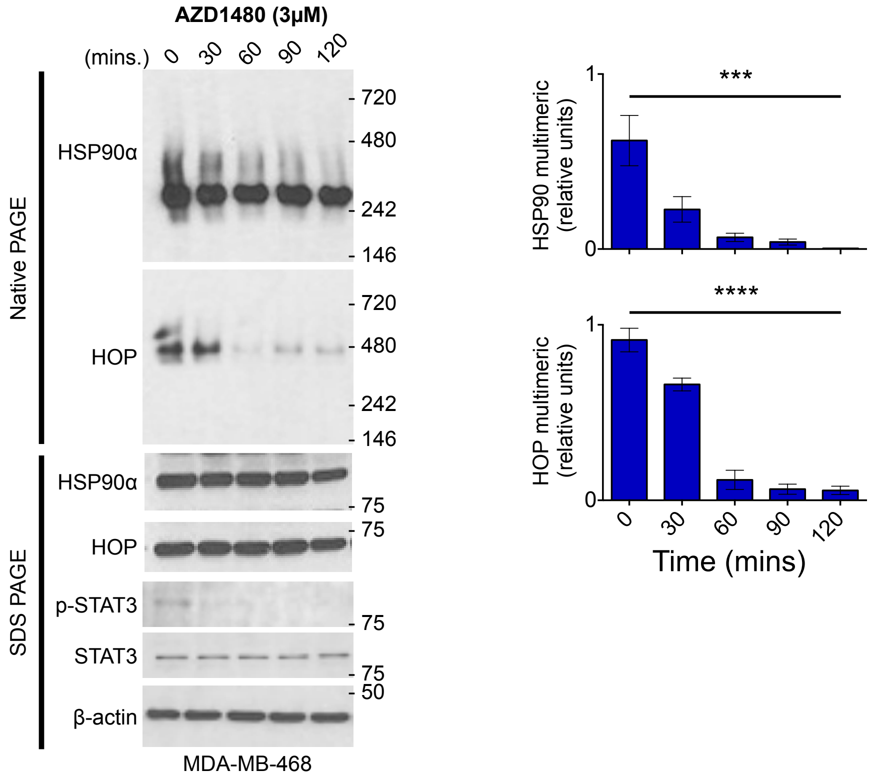

Supplementary Fig. 9. Interrelationship between the stress chaperome and proteome. Stable chaperome complexes and the total levels of the chaperome members were investigated by Native PAGE and SDS PAGE, as indicated, following treatment of cells with the JAK/STAT inhibitor AZD1480. Mean ± SEM, n = 3 individual values from the different experiments shown as points, One-Way ANOVA, **** = p<0.0001; *** = p<0.001.

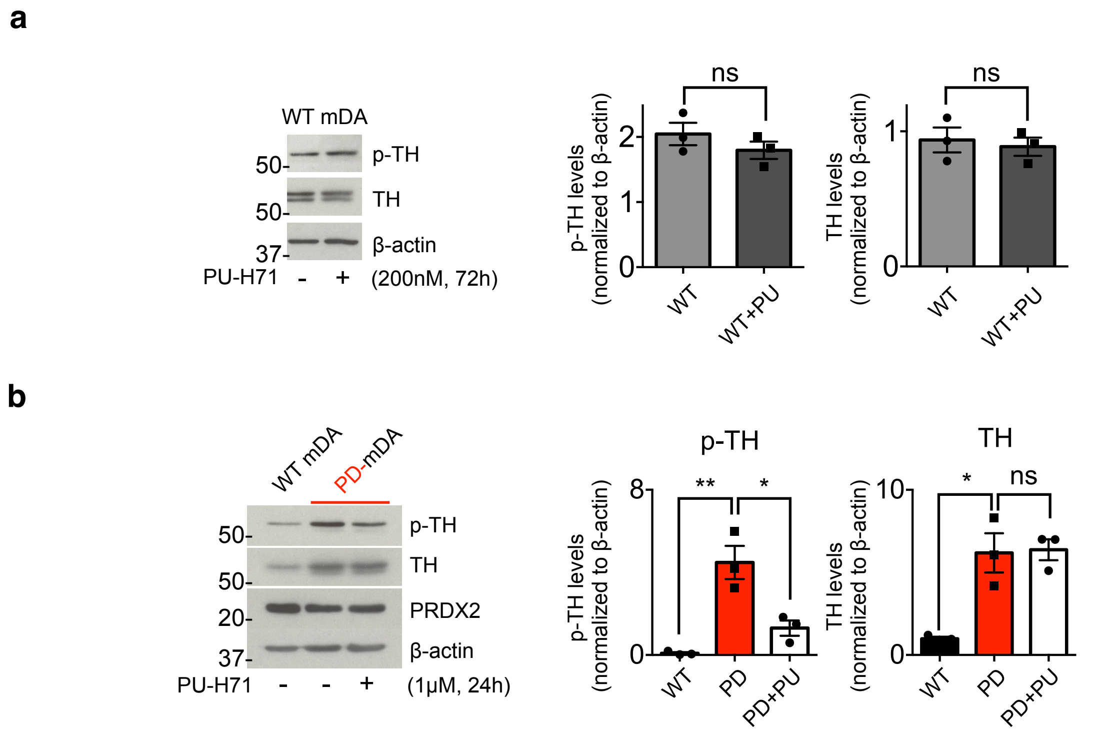

Supplementary Fig. 10. Tyrosine hydroxylase is regulated by the S-HSP90 networks specifically under PD-related stress. **a** Western blot analyses of chronic PU-H71 treatment (200nM for 72h) of mature WT mDA neurons. Mean ± SEM, n = 3 individual values from the different experiments shown as points, t-test, ns = p>0.05. **b** Western blot analyses of acute high-concentration PU-H71 treatment (1µM for 24h) on p-TH and TH in PD mDA neurons. WT mDA neurons are shown for comparison. PRDX2, control protein (i.e. S-HSP90 network regulated only under toxic stress conditions, see Fig. 5). Mean ± SEM, n = 3 individual values from the different experiments shown as points, t-test, ** = p<0.01; * = p<0.05; ns = p>0.05.

Antigen	Company, catalog number	Host	Dilution
AHA1	Abcam, ab56721	Mouse	1:1000 (WB)
CHIP	Cell Signaling, 2080	Rabbit	1:1000 (WB)
ERK1/2	Cell Signaling, 4695	Rabbit	1:1000 (WB)
P-ERK1/2 (T202/Y204)	Cell Signaling, 4377	Rabbit	1:1000 (WB)
FOXA2	Santa Cruz, SC-6554	Goat	1:200 (ICC)
P23	Abcam, ab2814	Mouse	1:1000 (WB)
HOP	Enzo Life Sciences, SRA-1500	Mouse	1:1000 (WB)
HSC70	Enzo Life Sciences, SPA-815	Rat	1:1000 (WB)
HSP40	Enzo Life Sciences, SPA-400	Rabbit	1:1000 (WB)
HSP60	Enzo Life Sciences, SPA-806	Mouse	1:1000 (WB)
HSP70	Enzo Life Sciences, SPA-810	Mouse	1:1000 (WB)
HSP90	Stressmarq, SMC-107	Mouse	1:1000 (WB)
LMX1A	Millipore, AB10533	Rabbit	1:2000 (ICC)
MAP2	Sigma, M1406	Mouse	1:200 (ICC)
NURR1	Perseus Proteomics, PP-N1404-00	Mouse	1:1000 (ICC)
GRP75	Stressmarq, SMC-133	Mouse	1:1000 (WB)
HSP110	Stressmarq, SPC-195	Rabbit	1:1000 (WB)
p-TH (S40)	Cell Signaling, 2791	Rabbit	1:1000 (WB)
p-STAT3 (Y705)	Cell Signaling, 9131	Rabbit	1:1000 (WB)
HSP90α	Abcam, ab2928	Rabbit	1:1000 (WB)
P70 S6 kinase	Cell Signaling, 2708	Rabbit	1:1000 (WB)
P-P70 S6 kinase, (T389)	Cell Signaling, 9234	Rabbit	1:1000 (WB)
S6	Cell Signaling, 2217	Rabbit	1:1000 (WB)
P-S6, (S235/236)	Cell Signaling, 4858	Rabbit	1:1000 (WB)
COX IV	Cell Signaling, 4850	Rabbit	1:1000 (WB)
TOM20	Cell Signaling, 42406	Rabbit	1:1000 (WB)
P65	Cell Signaling, 8242	Rabbit	1:1000 (WB)
P-P65	Cell Signaling, 3033	Rabbit	1:1000 (WB)
PRDX2	Sigma, WH0007001M1	Mouse	1:1000 (WB, ICC)
STAT3	Cell Signaling, 9139	Mouse	1:1000 (WB)
TH	Novus, NB-300-109	Rabbit	1:1000 (WB)
TH	Pel-Freez, P40101-150	Rabbit	1:500 (ICC, IHC)
TUJ1	Covance, MMS-435P	Mouse	1:500 (ICC)
β-ACTIN	Sigma, A1978	Mouse	1:2000 (WB)

Supplementary Table 1. Antibodies used in the study and their characteristics

Code	Age at onset	Age at biopsy taking	Clinical status	Gene	Mutation gene	Allele	Fibroblasts	Neurons
L3244	n/a	41	definite PD	*PARK2*	del Ex1+c. 924C>T	compound heterozygo us		x
B125-58	43	58	definite PD	*PARK2*	c.1072Tdel	homozygou s	x	x
B125-62	43	62	definite PD	*PARK2*	c.1072Tdel	homozygou s		x
L3035	31	49	definite PD	*PARK2*	delEx3-4 + duplEx7-12	compound heterozygo us	x	
L2122	61	69	definite PD	*PINK1*	c.1366C>T	homozygou s		x
L4993	NA	63	unaffected	Control			x	
L6396	NA	57	unaffected	Control			x	x
L2131	NA	59	unaffected	Control				x

Supplementary Table 2. Lines used in this study and their characteristics

Supplementary Methods. Synthesis and characterization of chemical biology tools

Supplementary Methods

The synthesis of chemical biology tools **PU-H71**, **PU-FITC**, **PU-alkyne** and **PU-bait** are shown in Scheme 1. **PU-H71**, **PU-FITC** and **PU-bait** were synthesized according to our previous publications.[1,2,5] Key intermediate **2**[1] was obtained from the 9-alkylation of 8-arylsulfanylpurine (**1**)[1] with 1,3-dibromopropane in 37% yield. **PU-H71**[1] and **PU-alkyne** were obtained from the amination of **2** with isopropylamine or 6-amino-1-hexyne (**3**) in 93% and 60% yield, respectively. **PU-FITC**[2] was obtained in 99% yield from the reaction of **PU-H71** with fluorescein isothiocyanate. Boc-protected amino purine **5**[5] was obtained in 90% yield from the reaction of **2** with *tert*-butyl 6-aminohexylcarbamate (**4**). Deprotection of **5** with TFA followed by reaction with Affi-Gel® 10 resulted in **PU-bait**.[5]

Scheme 1. Synthesis of PU-scaffold chemical biology tools

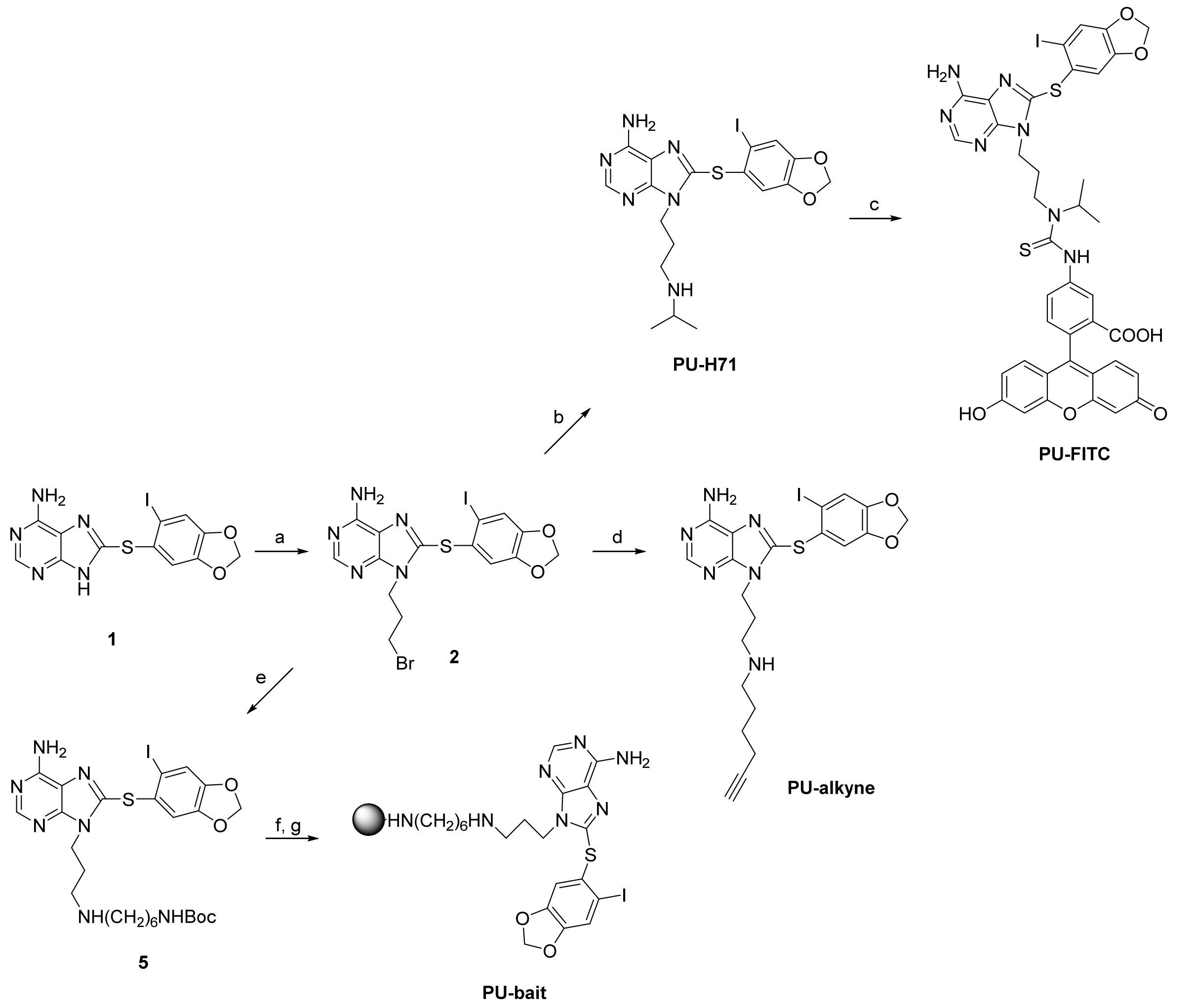

Reagents and conditions: (a) Cs_2CO_3, 1,3-dibromopropane, DMF, rt; (b) isopropylamine, DMF, rt; (c) FITC, Et_3N, DMF, rt; (d) 6-amino-1-hexyne (**3**), DMF, rt; (e) $NH_2(CH_2)_6NHBoc$ (**4**), DMF, rt; (f) TFA, CH_2Cl_2, rt; (g) Affi-Gel® 10, DIEA, DMAP, DMF.

PU-inert was used as an inactive control compound and was synthesized according to Scheme 2. Tosylation of 2-methoxyethanol (**6**) resulted in **7** in 66% yield, which was then refluxed in acetone with NaI to give 1-iodo-2-methoxyethane (**8**) in 54% yield. Thioether **9** was prepared from the reaction of 8-mercaptopurine and **8** with KOH in 87% yield. **9** was alkylated with 1,3-dibromopropane to give **10** in 28% yield, which was then reacted with 6-amino-1-hexyne (**3**) to give **PU-inert** in 63% yield.

Scheme 2. Synthesis of PU-inert

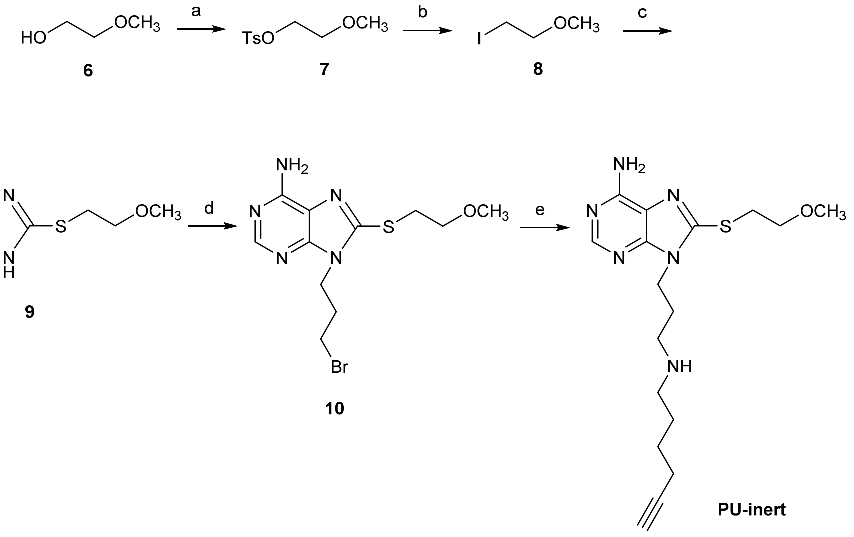

Reagents and conditions: (a) *p*-Tosylchloride, pyridine, 0°C, 2 h; (b) NaI, acetone, reflux, 12 h; (c) 8-mercaptopurine, 1.5 M KOH, rt, 24 h; (d) 1,3-dibromopropane, Cs_2CO_3, rt, 1 h; (e) 6-amino-1-hexyne (**3**), DMF, rt, overnight.

Chemistry

General Methods

^1H and ^{13}C NMR spectra were recorded on a Bruker 500 or 600 MHz instrument. Chemical shifts are reported in δ values in ppm downfield from TMS as the internal standard. ^1H data are reported as follows: chemical shift, multiplicity (s = singlet, d = doublet, t = triplet, q = quartet, br = broad, m = multiplet), coupling constant (Hz), integration. Low resolution mass spectra were obtained on a Waters Acquity Ultra Performance LC with electrospray ionization and SQ detector. High-performance liquid chromatography analyses were performed on a Waters Autopurification system with PDA, MicroMass ZQ and ELSD detector and a reversed phase column (Waters X-Bridge C18, 4.6 x 150 mm, 5 μm) using a gradient of (a) H_2O + 0.1% TFA and (b) CH_3CN + 0.1%

TFA, 5 to 95% b over 13 minutes at 1.2 mL/min. Column chromatography was performed using 230-400 mesh silica gel (EMD). Affi-Gel® 10 beads were purchased from Bio-Rad (Hercules, CA). Fluorescein isothiocyanate (FITC) was purchased from Aldrich. TMR-azide used in the CLICK assay was prepared as previously described.[6]

9-(3-Bromopropyl)-8-(6-iodo-benzo[1,3]dioxol-5-ylsulfanyl) adenine (2).[1] In a 250 mL RBF flushed with argon wrapped in aluminum foil, Cs_2CO_3 (4.73 g, 14.5 mmol, 1.2 eq.) and 1,3-dibromopropane (12.2 g, 6.2 mL 60.5 mmol, 5 eq.) was added to a solution of **1**[1] (5.0 g, 12.1 mmol) in anhydrous DMF (150 mL) at rt. After stirring for 45 min. additional Cs_2CO_3 (0.788 g, 2.42 mmol, 0.2 eq.) was added and it was stirred for an additional 45 min. Then the reaction mixture was filtered and DMF was removed under high vacuum (at or below 30 °C). The residue was purified by silica gel chromatography (CH_2Cl_2:MeOH:AcOH, 120:1:0.5 to 90:1:0.5). Fractions containing product were combined, concentrated under reduced pressure, then dried under high vacuum to a solid. This was evaporated from MeOH several times to give **2** (2.38 g, 37%). ^{1}H NMR (500 MHz, $CDCl_3$/MeOH-*d₄*) 8.24 (s, 1H); 7.38 (s, 1H), 7.04 (s, 1H), 6.05 (s, 2H), 4.37 (t, *J* = 7.1 Hz, 2H), 3.45 (t, *J* = 6.6 Hz, 2H), 2.41 (quin, *J* = 6.9 Hz, 2H); MS (ESI): *m/z* 533.9/535.9 $[M+H]^+$.

8-(6-Iodo-benzo[1,3]dioxol-5-ylsulfanyl)-9-(3-isopropylaminopropyl) adenine (PU-H71).[1] In a 1 L RBF flushed with argon and wrapped in aluminum foil, isopropylamine (79.7 g, 115 mL, 1.35 mol) was added to a solution of **2**[1] (14.4 g, 0.027 mol) in DMF (350 mL) at rt and was stirred overnight. The solvent was removed under vacuum and the crude solid was purified by silica gel chromatography (CH_2Cl_2:MeOH:MeOH-NH_3 (7N), 90:0.5:0.5 to 30:0:1). Fractions containing product were combined, concentrated and dried under high vacuum to give **PU-H71** (12.8 g, 93%). ^{1}H NMR (500 MHz, $CDCl_3$) δ 8.33 (s, 1H), 7.31 (s, 1H), 6.89 (s, 1H), 5.99 (s, 2H), 5.58 (br s, 2H), 4.30 (t, *J* = 7.0 Hz, 2H), 2.74-2.69 (m, 1H), 2.58 (t, *J* = 6.8 Hz, 2H), 2.01-1.96 (m, 2H), 1.03 (d, *J* = 6.2 Hz, 6H); MS (ESI): *m/z* 513.0 $[M+H]^+$.

PU-FITC.[2] **PU-H71**[1] (16.7 mg, 0.0326 mmol), FITC (14.0 mg, 0.0359 mmol) and Et_3N (0.1 mL) in DMF (0.2 mL) was stirred for 5 h at rt. The reaction mixture was concentrated under reduced pressure and the residue was purified by HPLC to give 21.2 mg (72%) of **PU-FITC**. ^{1}H NMR (500 MHz, $CDCl_3$) δ 8.15 (s, 1H), 7.86 (s, 1H), 7.77 (d, *J* = 7.9 Hz, 1H), 7.34 (s, 1H), 7.09 (d, *J* = 7.9 Hz, 1H), 7.01 (s, 1H), 6.63-6.71 (m, 4H), 6.51 (d, *J* = 7.3 Hz, 2H), 6.02 (s, 2H), 5.53 (br s, 2H), 4.30 (br s, 2H), 3.64 (br s, 2H), 2.85 (br s, 1H), 2.27 (m, 2H), 1.23 (d, *J* = 6.2 Hz, 6H); HRMS (ESI) *m/z* $[M+H]^+$ calcd. for $C_{39}H_{33}IN_7O_7S_2$, 902.0928; found 902.0942; HPLC: t_R = 9.90 (99%).

6-Amino-1-hexyne (3).[3] **3** was prepared by a previously descibed procedure with some modification.[3] Hydrazine hydrate (1.1 mL, 22 mmol) was added to a fine suspension of N-(5-hexynyl)phthalimide (1 g, 4.4 mmol) in methanol (25 mL). The resulting solution was stirred at rt for overnight. Next day, additional methanol (50 mL) was added to the reaction mixture and solids filtered. The filtrate was concentrated under reduced pressure and the resulting residue purified by column chromatography (CH_2Cl_2:MeOH-NH_3 (7N), 50:1 to 10:1) to give 0.383 g (89%) of **3**. MS (ESI) *m/z* 97.8 $[M+H]^+$.

9-(3-(Hex-5-yn-1-ylamino)propyl)-8-((6-iodobenzo[d][1,3]dioxol-5-yl)thio)-9H-purin-6-amine (PU-alkyne). A mixture of **2**[1] (21.8 mg, 0.041 mmol) and **3**[3] (200 mg, 2.04 mmol) in DMF (1 mL) was stirred at room temperature under nitrogen for overnight. Solvent was removed under reduced pressure and the resulting residue was purified by preparatory TLC (CH_2Cl_2:MeOH-NH_3 (7N), 15:1) to give 13.6 mg (60%) of **PU-alkyne**. ^{1}H NMR (600 MHz, $CDCl_3$/MeOH-*d₄*) δ 8.14 (s, 1H), 7.31 (s, 1H), 6.97 (s, 1H), 5.98 (s, 2H), 4.22 (t, *J* = 6.9 Hz, 2H), 2.49-2.61 (m, 4H), 2.15 (td, *J* = 7.0, 2.6 Hz, 2H), 1.95-2.04 (m, 2H), 1.92 (t, *J* = 2.6 Hz, 1H), 1.55-1.62 (m, 2H), 1.45-1.54 (m,

2H); ^{13}C NMR (150 MHz, $CDCl_3$/MeOH-*d_4*) δ 154.2, 152.3, 151.3, 149.8, 149.4, 147.5, 125.6, 119.5, 119.3, 113.8, 102.6, 94.2, 84.0, 68.6, 45.7, 41.2, 29.0, 28.2, 26.0, 18.1; HRMS (ESI) *m/z* $[M+H]^+$ calcd. for $C_{21}H_{24}IN_6O_2S$, 551.0726; found 551.0712.

***tert*-Butyl 6-aminohexylcarbamate (4).**[4] 1,6-diaminohexane (10 g, 0.086 mol) and Et_3N (13.05 g, 18.13 mL, 0.129 mol) were suspended in CH_2Cl_2 (300 mL). A solution of di-*tert*-butyl dicarbonate (9.39 g, 0.043 mol) in CH_2Cl_2 (100 mL) was added dropwise over 90 minutes at rt and stirring continued for 18 h. The reaction mixture was added to a separatory funnel and washed with water (100 mL), brine (100 mL), dried over Na_2SO_4 and concentrated under reduced pressure. The resulting residue was chromatographed (CH_2Cl_2:MeOH-NH_3 (7N), 70:1 to 20:1) to give 7.1 g (76%) of **4**. ^1H NMR ($CDCl_3$) δ 4.50 (br s, 1H), 3.11 (br s, 2H), 2.68 (t, *J* = 6.6 Hz, 2H), 1.44 (s, 13H), 1.33 (s, 4H); MS (ESI): *m/z* 217.2 $[M+H]^+$.

***tert*-Butyl 6-(3-(6-amino-8-(6-iodobenzo[d][1,3]dioxol-5-ylthio)-9H-purin-9-yl)propylamino)hexylcarbamate (5).**[5] **2** (0.226 g, 0.423 mmol) and **4** (0.915 g, 4.23 mmol) in DMF (7 mL) was stirred at rt for 24 h. The reaction mixture was concentrated and the residue chromatographed ($CHCl_3$:MeOH:MeOH-NH_3 (7N), 100:7:3) to give 0.255 g (90%) of **5**. ^1H NMR ($CDCl_3$) δ 8.32 (s, 1H), 7.31 (s, 1H), 6.89 (s, 1H), 5.99 (s, 2H), 5.55 (br s, 2H), 4.57 (br s, 1H), 4.30 (t, *J* = 7.0 Hz, 2H), 3.10 (m, 2H), 2.58 (t, *J* = 6.7 Hz, 2H), 2.52 (t, *J* = 7.2 Hz, 2H), 1.99 (m, 2H), 1.44 (s, 13H), 1.30 (s, 4H); HRMS (ESI) *m/z* $[M+H]^+$ calcd. for $C_{26}H_{37}IN_7O_4S$, 670.1673; found 670.1670; HPLC: t_R = 7.02 min.

PU-H71-Affi-Gel® 10 beads (PU-bait).[5] **5** (0.301 g, 0.45 mmol) was dissolved in 15 mL of CH_2Cl_2:TFA (4:1) and the solution was stirred at rt for 45 min. Solvent was removed under reduced pressure and the residue dried under high vacuum overnight. This was dissolved in DMF (12 mL) and added to 25 mL of Affi-Gel® 10 beads (prewashed, 3 x 50 mL DMF) in a solid phase peptide synthesis vessel. 225 µL of N,N-diisopropylethylamine and several crystals of DMAP were added and this was shaken at rt for 2.5 h. Then 2-methoxyethylamine (0.085 g, 97 µl, 1.13 mmol) was added and shaking was continued for 30 minutes. Then the solvent was removed and the beads washed for 10 minutes each time with CH_2Cl_2:Et_3N (9:1, 4 x 50 mL), DMF (3 x 50 mL), Felts buffer (3 x 50 mL) and *i*-PrOH (3 x 50 mL). The beads (**PU-bait**) were stored in *i*-PrOH (beads: *i*-PrOH (1:2), v/v) at -80 °C.

2-Methoxyethyl 4-methylbenzenesulfonate (7).[7] A mixture of *p*-toluenesulfonylchloride (5.26 g, 0.0276 mmol) and pyridine (5 mL) is stirred under argon at 0 °C in an ice-water bath. Then 2-methoxyethanol (**6**; 2.07 mL, 0.0263 mmol) is added slowly and stirred at 0 °C for 2 h. Then the reaction mixture was diluted with ice-cold dH_2O (40 mL) and extracted with CH_2Cl_2 (2 x 40 mL). The combined organic layers were washed with ice-cold 6M HCl (2 x 15 mL) and dH_2O (15 mL), dried over $MgSO_4$, filtered and concentrated to give 3.98 g (66%) of **7**, which was used directly in the next step without any further purificaion. ^1H NMR (500 MHz, $CDCl_3$) δ 7.81 (d, *J* = 8.3 Hz, 2H), 7.34 (d, *J* = 8.1 Hz, 2H), 4.16 (t, *J* = 4.7 Hz, 2H), 3.58 (t, *J* = 4.8 Hz, 2H), 3.31 (s, 3H), 2.45 (s, 3H).

1-Iodo-2-methoxyethane (8).[7] A mixture of **7** (3.98 g, 0.017 mmol) and NaI (7.88 g, 0.0526 mmol) in acetone (40 mL) was refluxed for 12 h. Then the reaction mixture was filtered over celite and concentrated under reduced pressure. The resulting residue was taken up into CH_2Cl_2 (100 mL), washed with 10% sodium thiosulfate (2 x 25 mL), 5% $NaHCO_3$ (2 x 50 mL) and dH_2O (2 x 50 ml), dried over $MgSO_4$, filtered and concentrated to give 1.73 g (54%) of **8**, which was used directly in the next step without any further purification. ^1H NMR (500 MHz, $CDCl_3$) δ 3.66 (t, *J* = 6.6 Hz, 2H), 3.40 (s, 3H), 3.26 (t, *J* = 6.7 Hz, 2H); ^{13}C NMR (125 MHz, $CDCl_3$) δ 73.1, 58.5, 2.8.

8-((2-Methoxyethyl)thio)-9H-purin-6-amine (9). A mixture of 8-mercaptopurine[1] (0.441 g, 2.64 mmol) and **8** (0.573 g, 3.08 mmol) in 1.5 M KOH (*aq.*) (1.1 mL) was stirred at rt for 24 h. The reaction mixture was concentrated under reduced pressure and the resulting residue purified by ISCO (CH_2Cl_2:MeOH, 0 to 15% MeOH) to give 0.52 g (87%) of **9**. ^{1}H NMR (600 MHz, DMSO-d_6) δ 13.04 (br s, 1H), 8.04 (s, 1H), 7.07 (br s, 2H), 3.61 (t, *J* = 6.3 Hz, 2H), 3.44 (t, *J* = 6.2 Hz, 2H), 3.27 (s, 3H); MS (ESI) *m/z* 226.0 $[M+H]^+$.

9-(3-Bromopropyl)-8-((2-methoxyethyl)thio)-9H-purin-6-amine (10). A mixture of **9** (0.250 g, 1.11 mmol), 1,3-dibromopropane (567 µL, 5.55 mmol) and Cs_2CO_3 (0.542 g, 1.67 mmol) in DMF (11 mL) was stirred at rt for 1 h. It was concentrated under reduced pressure and purified by column chromatography (CH_2Cl_2:MeOH:AcOH, 150:1:0.5 to 50:1:0.5) to give 0.108 g (28%) of **10**. ^{1}H NMR (500 MHz, $CDCl_3$) δ 8.22 (s, 1H), 5.91 (br s, 2H), 4.27 (t, *J* = 7.0 Hz, 2H), 3.75 (t, *J* = 6.1 Hz, 2H), 3.56 (t, *J* = 6.0 Hz, 2H), 3.38-3.45 (m, 5H), 2.36-2.45 (m, 2H); MS (ESI) *m/z* 346.0/348.0 $[M+H]^+$.

9-(3-(Hex-5-yn-1-ylamino)propyl)-8-((2-methoxyethyl)thio)-9H-purin-6-amine (PU-inert). A mixture of **10** (31 mg, 0.0895 mmol) and 6-amino-1-hexyne (**3**; 383 mg, 3.9 mmol) in DMF (1 mL) was stirred at rt under nitrogen for overnight. Solvent was removed under reduced pressure and the resulting residue was purified by preparatory TLC (CH_2Cl_2:MeOH-NH_3 (7N), 10:1) to give 20.4 mg (63%) of **PU-inert**. ^{1}H NMR (600 MHz, $CDCl_3$) δ 8.19 (s, 1H), 5.64 (br s, 2H), 4.14 (t, *J* = 7.0 Hz, 2H), 3.66 (t, *J* = 5.1 Hz, 2H), 3.48 (t, *J* = 6.1 Hz, 2H), 3.33 (s, 3H), 2.48-2.55 (m, 4H), 2.14 (td, *J* = 6.9, 2.6 Hz, 2H), 1.91-1.99 (m, 2H), 1.89 (t, *J* = 2.6 Hz, 1H), 1.46-1.58 (m, 4H); ^{13}C NMR (150 MHz, $CDCl_3$) δ 152.3, 151.2, 150.7, 148.6, 118.7, 83.3, 69.8, 67.5, 57.8, 48.3, 45.2, 39.7, 30.7, 28.4, 28.0, 25.2, 17.3; HRMS (ESI) *m/z* $[M+H]^+$ calcd. for $C_{17}H_{27}N_6OS$, 363.1967; found 363.1958.

Supplementary References

1. He, H.; Zatorska, D.; Kim, J.; Aguirre, J.; Llauger, L.; She, Y.; Wu, N.; Immormino, R. M.; Gewirth, D. T.; Chiosis, G., *J. Med. Chem.* **2006**, 49, 381-390.
2. Taldone, T.; Gomes-DaGama, E.M.; Zong, H.; Sen, S.; Alpaugh, M.L.; Zatorska, D.; Alonso-Sabadell, R.; Guzman, M.L.; Chiosis G., *Bioorg. Med. Chem. Lett.* **2011**, 21, 5347-5352.
3. Rozkiewicz, D. I.; Janczewski, D.; Verboom, W.; Ravoo, B. J.; Reinhoudt, D. N. *Angew. Chem., Int. Ed.* **2006**, *45*, 5292-5296.
4. Hansen, J. B.; Nielsen, M. C.; Ehrbar, U.; Buchardt, O., *Synthesis* **1982**, 404-405.
5. Taldone, T.; Zatorska, D.; Patel, P.D.; Zong, H.; Rodina, A.; Ahn, J.H.; Moulick, K.; Guzman, M.L.; Chiosis, G., *Bioorg. Med. Chem.* **2011**, 19, 2603-2614.
6. Salic, A.; Mitchison, T. J. *Proc. Natl. Acad. Sci. U S A* **2008**, *105*, 2415-2420.
7. Vennemann, M.; Baer, T.; Gruenewald, S.; Boehm, M.; Fuegedi, P. **2009**, *4SC A.-G. Germany, WO 2009024613*.
