# Supplementary material for: HSP90-incorporating chaperome networks as biosensor for disease-related pathways in patient-specific midbrain dopamine neurons
Source: Nat Commun. 2018 Oct 19;9:4345. doi: 10.1038/s41467-018-06486-6 (PMC6195591; doi:10.1038/s41467-018-06486-6)
Supplement: Supplementary file 2 — Description of Additional Supplementary Files [file 41467_2018_6486_MOESM2_ESM.pdf]

Description of Additional Supplementary Files:

Supplementary Data 1. Interactome Datasets. Interactome Datasets identified using spectral counting (sheet 1), LFQ (sheet 2) and SILAC (sheet 3) and UNIPROT and GO annotations

Supplementary Data 2. Statistical analyses and protein enrichment determinations for each of the PD related stresses. Statistical analyses over the ESC-identified interactome datasets, sheets 1 through 5. Sheet 1, PD (PARKIN) vs WT; sheet 2, WT vs WT + CCCP; sheet 3, WT vs WT + Rotenone; sheet 4, PD vs PD + CCCP; sheet 5, PD vs PD + Rotenone. Sheets 6 through 10, proteins enriched in each of the stress conditions as calculated via methods described in sheets 1 through 5. Sheet 11, Statistical analyses over the LFQ-identified interactome datasets, WT versus PD. Sheets 12, proteins enriched in each of the stress conditions as calculated via methods described in sheet 11. Sheet 13, comparison of the LFQ and ESC-identified interactomes (see Supplementary Fig. 6b for the Venn diagram representation of the data)

Supplementary Data 3. Reactome analyses over the identified interactomes. Sheet 1, description of datasets included in sheets 2 through 8. Sheet 2 through 4, interactome datasets selected based on the statistical analyses described in Supplementary Data 2. Sheets 5 through 7, Reactome pathway analyses over the interactome datasets listed in sheets 2 through 4. Sheets 8, Reactome pathway analyses over the LFQ identified interactome, enriched in PD over WT.

Supplementary Data 4. GO enrichment analyses over the identified interactomes. Sheet 1, contingency table; sheet 2, full GO enrichment analysis
